# Atomic-Scale Characterization of Microscale Battery Particles Enabled by a High-Throughput Focused Ion Beam Milling Technique

**DOI:** 10.1021/acsomega.4c00318

**Published:** 2024-04-06

**Authors:** Alexi L. Pauls, Melissa J. Radford, Audrey K. Taylor, Byron D. Gates

**Affiliations:** Department of Chemistry, Simon Fraser University, 8888 University Drive, Burnaby, British Columbia V5A 1S6, Canada

## Abstract

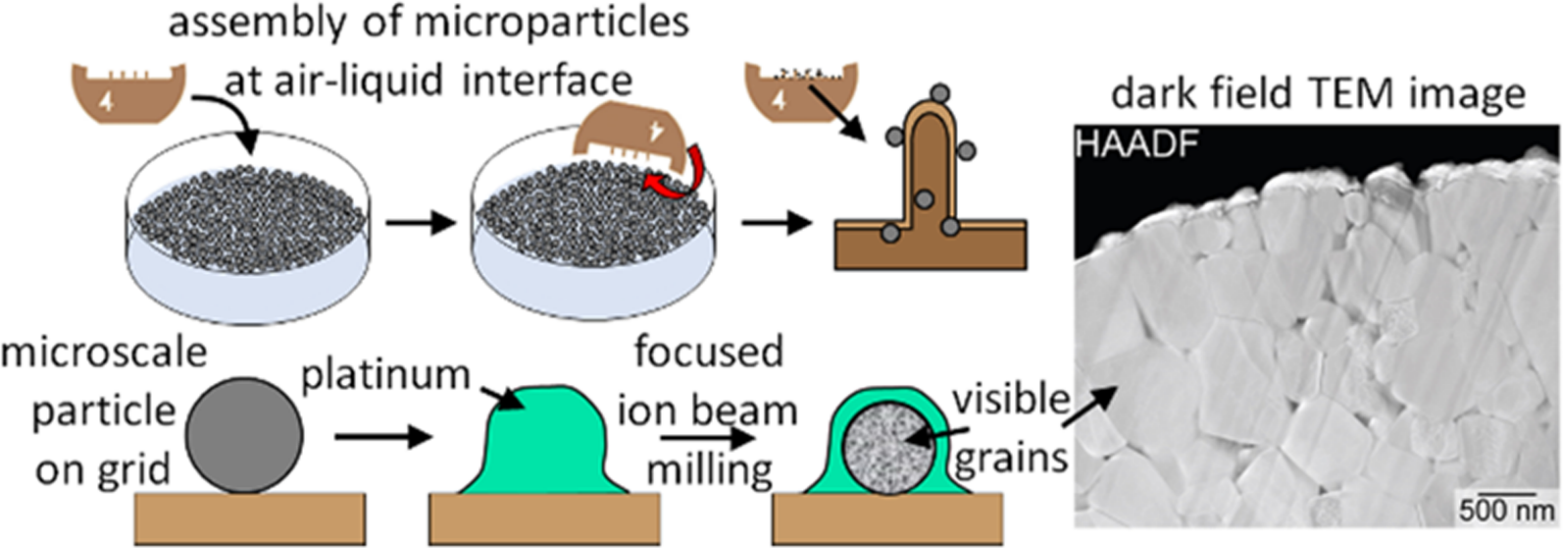

The cathode materials in lithium-ion batteries (LIBs) require improvements to address issues such as surface degradation, short-circuiting, and the formation of dendrites. One such method for addressing these issues is using surface coatings. Coatings can be sought to improve the durability of cathode materials, but the characterization of the uniformity and stability of the coating is important to assess the performance and lifetime of these materials. For microscale particles, there are, however, challenges associated with characterizing their surface modifications by transmission electron microscopy (TEM) techniques due to the size of these particles. Often, techniques such as focused ion beam (FIB)-assisted lift-out can be used to prepare thin cross sections to enable TEM analysis, but these techniques are very time-consuming and have a relatively low throughput. The work outlined herein demonstrates a FIB technique with direct support of microscale cathode materials on a TEM grid that increases sample throughput and reduces the processing time by 60–80% (i.e., from >5 to ∼1.5 h). The demonstrated workflow incorporates an air–liquid particle assembly followed by direct particle transfer to a TEM grid, FIB milling, and subsequent TEM analysis, which was illustrated with lithium nickel cobalt aluminum oxide particles and lithium manganese nickel oxide particles. These TEM analyses included mapping the elemental composition of cross sections of the microscale particles using energy-dispersive X-ray spectroscopy. The methods developed in this study can be extended to high-throughput characterization of additional LIB cathode materials (e.g., new compositions, coating, end-of-life studies), as well as to other microparticles and their coatings as prepared for a variety of applications.

## Introduction

The choice of cathode materials in lithium-ion batteries (LIBs) is well understood to have an impact on cell durability and performance, but precise characterization techniques are necessary to quantify these changes at an atomic level.^1−3^ Improvements to cathode materials for LIBs can include tuning the ratio of metal species therein, creating mixed composites, and implementing of surface modifications or protective coatings.^4−8^ For example, cathode materials can be tuned to achieve different energy densities by adjusting their composition or surface coatings of a nanoscale thickness can be applied to improve stability to electrochemical cycling.^9,10^ Surface coatings are of particular interest due to their ability to improve the structural and thermal stabilities of cathode materials. They can also provide benefits that include adjusting interlayer lattice spacing to enhance lithium ion (Li^+^) migration, in addition to serving as a protective layer against cathode degradation by preventing negative interactions between the inner cathode material and surrounding electrolyte (e.g., preventing dendrite formation and metal ion dissolution, minimizing solid-electrolyte interface formation).^11−16^ Other methods for improving the Li^+^ diffusion include the use of tuning the structures at the interfaces of these cathode particles, such as changing the porosity of the material or tuning of its crystalline facets.^2,17−21^ It is of the utmost importance to sufficiently characterize the cathode materials and modifications to these materials, such as the application of nanoscale thick surface coatings, increasing the porosity, or tuning crystalline facets, to draw appropriate conclusions with regard to changes observed in their performance as a component of LIBs. Commonly used material characterization techniques for cathode materials in LIBs include X-ray diffraction to assess their crystallinity and transmission electron microscopy (TEM) to assess their nanoscale features and their composition. In addition, the use of in situ or operando TEM techniques can be used for material characterization under operating conditions.^22^ A primary challenge with utilizing TEM techniques is the thickness of the sample, which must be sufficiently thin for electrons to be transmitted through the sample (e.g., nominally ≤100 nm).^23,24^ Due to this size restriction, the analysis of microscale particles—where the particle diameter is >1 μm—by TEM is challenging due to the inability of electrons to be transmitted through the sample. For sufficiently small materials (e.g., ≤ 100 nm in diameter), a TEM analysis may be performed without further modifications to these materials.^25−28^ Additional techniques, such as focused ion beam (FIB)-assisted lift-out or ultramicrotome, are often used prior to TEM analysis to prepare sufficiently thin cross sections of the desired materials. Whether the modifications made to cathode materials to improve their performance and durability are on the microscale (e.g., structural designs) or the nanoscale (e.g., thin coatings), it is essential to characterize the uniformity, composition, and crystallinity of these features.

Cross sections of microscale or larger samples can be prepared using FIB milling followed by a FIB-assisted lift-out of these thin sections for analysis by scanning electron microscopy (SEM), FIB-SEM, or TEM techniques. In FIB methods, a dual-beam electron microscope is used to prepare the thin section, image, and guide the lift-out. A beam of high-energy electrons is used for imaging the sample and to guide the lift-out process, and a second beam composed of gallium ions (Ga^+^) is used for the FIB processes.^29,30^ The Ga^+^ beam is used to selectively remove portions of the material from the sample to create a thin cross section. This thin section of the sample is subsequently cut away from the rest of the sample by FIB techniques and can be imaged within the dual-beam system by SEM methods or placed onto a sample support (e.g., a half-moon TEM grid) for further analysis by TEM techniques. Samples prepared by FIB preparation techniques (i.e., FIB cross sections or FIB lift-out) can be imaged by additional SEM-based techniques [e.g., electron backscatter diffraction (EBSD) and transmission Kikuchi diffraction (TKD)].^31−33^ Elemental analysis through energy-dispersive X-ray spectroscopy (EDS) using SEM methods can be challenging due to the interaction volume of electrons with the sample leading to relatively poor spatial resolution.^34^ These effects can be minimized by performing a full lift-out of a section of the sample for analysis by TEM techniques. A FIB-assisted lift-out procedure involves the preparation of the thin lamella (or section of the sample), which is subsequently welded to a micromanipulator needle by site-directed, selective deposition of a Pt-containing film. The lamella is cut free from the substrate by using FIB milling to enable the micromanipulator needle to transfer the lamella to the new substrate (e.g., half-moon TEM grid). The transfer is considered complete when the lamella has been subsequently welded onto the new substrate and cut free by FIB milling from the micromanipulator needle. To finalize the FIB-assisted lift-out process, the attached lamella must be carefully thinned to achieve a thickness of ∼100 nm. Supporting this thin section on a TEM grid enables its ease of transfer to a TEM system for subsequent high-resolution analyses (e.g., imaging and elemental mapping).

The use of Ga^+^ during the milling process can, however, result in damage to the sample by inducing amorphization in the outer layers or by implanting Ga^+^ in the sample.^35^ A few solutions have been developed to address damage caused by the Ga^+^ beam. The energy of the ion beam can be reduced with a corresponding decrease in the rate of sample milling and a decrease in resolution during FIB-assisted imaging.^36,37^ Lower energies are recommended for the final steps of FIB milling after large portions of the material have been removed using the high-energy Ga^+^ beam.^36^ In addition, the use of a two-step process to prepare a protective layer—where a thin layer of platinum (Pt) is first deposited with the assistance of the electron beam and subsequently additional material is deposited by the ion beam—can minimize the effects of Ga^+^-induced damage to the sample.^37^ The FIB-assisted lift-out has been used to prepare thin sections of the cathode materials of LIBs such as coated lithium manganese nickel cobalt oxide particles, which enabled a high-resolution imaging and elemental analysis of these core–shell particles by TEM techniques.^38^ The use of FIB-assisted lift-out techniques has also been used to study the formation of intergranular cracks and changes to the composition of microstructures as a result of delithiation processes.^39−41^ It is, however, desirable to utilize techniques that are less expensive and less time-consuming and that have a higher throughput than performing FIB-assisted lift-out of cathode materials to assess their nanoscale composition and structure.

Ultramicrotome techniques offer a relatively high-throughput method for preparing cross sections of samples that are sufficiently thin to be imaged directly by TEM techniques. In the ultramicrotome method, microscale particles can be embedded in an epoxy-based matrix that is cross-linked and subsequently sliced into thin sections using a diamond knife.^42,43^ Afterward, the thinly sliced cross sections are supported on a grid for analysis by TEM techniques. Ultramicrotome methods can provide a higher-throughput approach to preparing thin cross sections of a sample than FIB-assisted lift-out methods. Microtome techniques can prepare cross sections of up to hundreds of microscale particles per sample, but this method is indiscriminate in the particles to be analyzed, whereas in FIB-assisted techniques, particular particles can be selected for analysis and the cross section can be precisely defined relative to the particle itself. The ultramicrotome cutting procedure used to prepare cross sections can cause mechanical damage to the sample (i.e., fragmentation) and can result in the formation of a series of cross sections with varying degrees of thickness. These issues are especially prevalent in relatively hard materials.^43^ In addition, during the sectioning procedure, particles can become dislodged from the epoxy matrix upon contact with the knife edge if the bonds within the particle are stronger than their interactions with the encapsulating epoxy.^43^ Another challenge of using ultramicrotome techniques is the process of embedding the sample into the epoxy matrix, which can introduce contamination (e.g., carbon-based species) to the sample during TEM imaging.^43^ Ultramicrotome can be applied to sectioning some samples without the use of epoxy embedding, but this may present a challenge for sectioning highly porous materials since the epoxy can help preserve the structure of the material during the cutting process.^44^ Cross sections of LIB materials that have been prepared by ultramicrotome techniques include core–shell particles, layered structures, and doped microparticles.^42,45−47^ The use of ultramicrotome techniques to prepare cross sections has enabled a high-throughput investigation of multiple cross sections and the visualization of the outer layers of microscale cathode particles.^42^ Further analysis of cross sections prepared by ultramicrotome techniques can include elemental maps and an assessment of local changes in sample crystallinity by TEM techniques, but the diamond knife used to create these cross sections can sometimes introduce mechanical damage to these samples. While ultramicrotome techniques enable fast, high-throughput sample preparation, they lack the single-particle resolution of FIB-assisted lift-out procedures (i.e., microtome is nonspecific to the selection of individual microscale particles and the orientation along which they are sectioned). An alternative approach is sought to prepare thin cross sections of microscale materials that enable a detailed analysis of single particles through FIB-assisted lift-out procedures but without the time-consuming process required to achieve these cross sections and that could introduce less structural damage compared to using ultramicrotome techniques.

In this work, we demonstrate a method to prepare thin cross sections of microscale particles for detailed characterization by TEM of the composition and features within single particles. A film of microscale particles was initially assembled at an air–liquid interface and transferred onto a half-moon TEM grid by a dip coating process. After solvent evaporation, particles located at the edges of the TEM grid were imaged by SEM, and then a series of particles were selected for protection with a thin layer of platinum (Pt) and subsequent thinning using FIB techniques. This procedure offers a relatively fast and simple alternative to prepare nanoscale thin sections of microparticles without mechanical damage associated with ultramicrotome procedures. Moreover, the method enables high-resolution single-particle analysis, which does not require the use of epoxy materials that alter the interfaces of the particles by embedding the samples during the preparation of their cross sections. The procedure introduced herein was performed using commercially available microscale particles of lithium nickel cobalt aluminum oxide (NCA). These NCA particles were chosen as the primary material in this study to demonstrate this workflow due to the regular, spherical form of these particles and their relatively uniform size distribution, which assisted in the development of the procedures due to their clearly defined grain boundaries, distinct shape, and consistent size. The relatively high-throughput methods in this work to prepare nanoscale cross sections of microscale particles can be extended to the characterization of other materials, including other LIB cathode materials, such as lithium manganese nickel oxide (LMNO), lithium iron phosphate (LFP), lithium nickel manganese cobalt oxide (NMC), and other novel LIB cathode compositions and coatings. Further, these methods can be used to investigate recovered particles at the end of life for analysis of failure mechanisms in future works. In addition to the field of LIB, this FIB procedure can be extended to other microscale particles in diverse fields, including those used in biomedical applications.^48,49^ The FIB procedure developed in this work allowed for a 5× higher-throughput analysis of microscale LIB cathode materials compared to FIB-assisted lift-out, which can further be used to develop statistical analysis of individual microscale particles (e.g., preferential coating on selective crystalline facets, degradation mechanisms at end of life, reproducibility of synthesis methods).

## Results and Discussion

### Selection of Microscale Particles

The methods demonstrated in this study were initially developed by using commercially available NCA particles. These microscale particles are agglomerates of smaller particles and cannot be imaged directly by TEM techniques without further modifications, such as by using methods to prepare sufficiently thin cross sections for analyses. These NCA particles exhibit a number of properties that were sought to demonstrate the benefits of utilizing a faster and simpler method of preparing nanoscale cross sections of microscale particles through the methods outlined herein. For example, the NCA microscale particles are prepared from the assembly of primary nanoscale particles into a larger secondary particle. The packing structure of these primary particles is of interest as it relates to the energy density of these materials. For example, voids within the secondary particles that form between the assembled primary particles can reduce the overall energy density of these materials. The primary particles are crystalline, which provide clear grain boundaries and a distinct texture within the secondary particles. An analysis of the nanoscale cross sections of these secondary NCA particles could elucidate the presence of voids, impurities, and other defects that would impact their electrochemical performance. An investigation of the composition, structure, and texture within the secondary particles could also demonstrate whether there is a preferred orientation of the primary particles. Although not anticipated for the NCA particles, the orientation of some materials can be influenced by the oriented attachment of crystalline primary particles that lead to an overall preferred orientation within the secondary particle.^50,51^ The organization of the primary particles within the larger, microscale particle could have implications on the Li^+^ transport to and from these materials.^52−54^ In addition, depending on the methods used to prepare the primary particles of NCA, there could be variations in composition between each of the primary particles. Again, a method that enables an analysis of the variations down to the nanoscale within these microscale particles would provide feedback on the techniques used to prepare these and other types of cathode materials. These NCA particles are just one example of cathode materials composed of microscale, secondary particles (e.g., others include NMC 111 and NMC 811).^55−57^ The uniform, spherical shape of these NCA particles enabled the ease of selecting single particles for further analysis. Although our work used NCA materials, the composition of the NCA particles can also be modified to further improve their stability. For example, it has been reported that yttrium-doped zirconia-modified NCA particles demonstrate enhanced stability toward electrochemical cycling in comparison to pristine NCA particles, which otherwise exhibited a relatively high number of cracks within their secondary particles after repeated cycling.^58^ The methods developed in this study can be extended beyond the analysis of NCA materials to investigate compositional uniformity and structural changes within other types of microscale particles including NMC and other types of cathode materials for LIBs.

### Preparing Assemblies of Microscale Particles at an Air–Liquid Interface

A primary goal of our work was to facilitate faster throughput for sample preparation. The FIB-assisted lift-out process can provide access to nanoscale cross sections of samples. Much of the development work for FIB-assisted lift-out procedures has been aimed at semiconductor materials.^29,36,59,60^ Some work has extended these techniques to the characterization of cathode materials for LIBs.^39−41,61−64^ Preparing cross sections by FIB techniques (e.g., for SEM analysis) can be relatively fast, but the subsequent lift-out assisted procedure to prepare a lamella for TEM analysis is a time-consuming process and is prone to the risk of losing the sample. Pathways for loss of the sample include electrostatic charging of the sample during its transfer to the TEM grid and mechanical failure of the interface between the FIB-prepared cross section and the TEM grid during sample handling.^65−67^ Adhesion to the TEM grid is dependent on the surface area in contact between the particle and the grid, which is dependent on the particle size and shape. The contact can be facilitated by an in situ, FIB-assisted deposition of an adhesion layer as part of the lift-out procedure. The lift-out process itself could be bypassed if the particles were supported directly on the TEM grid. Half-moon-shaped TEM grids, prominently used in FIB lift-out procedures, were repurposed for directly supporting the microscale particles of interest. These grids are composed of solid copper (Cu) and have a series of protruding prongs for supporting samples while also providing an electron transparent background to the supported samples in comparison to more conventional TEM grids that contain a solid or porous support material (e.g., Formvar or lacey carbon supports).

It was necessary to establish the best method for applying the microscale cathode particles to the half-moon grids while achieving sufficient separation between the secondary particles to enable a single-particle analysis. The method was selected, in part, to prevent the formation of clusters of particles that would occlude the electron beam during TEM analyses. These clusters could also create instabilities such as when electrostatic charges build up between these secondary particles and the TEM grid during sample handling and TEM-based analysis. Ideally, the secondary particles would be positioned along the edges of the TEM grid as the grid itself was not electron transparent (e.g., 35 μm thick Cu support). It was also ideal that the particles were located along the edges of the “fingers” or prongs of the half-moon TEM grid for ease of preparing thin sections of the particles by FIB techniques.

A series of methods were evaluated for loading the NCA particles onto half-moon grids. These methods included drop-casting of the particles from a liquid suspension and dip coating the grid into a solution of particles. Drop-casting a sample onto a TEM grid from a colloidal suspension is a commonly used method to prepare TEM samples. In this series of tests, we found that drop-casting the NCA particles from suspensions resulted in the formation of clusters of particles spread across the surfaces of the TEM grid (Figure S1). These piles of particles resulted from the effects of capillary forces during solvent evaporation. In addition to the challenges outlined above for analyzing the clusters of particles, these clusters were often not appropriately positioned for analysis by TEM techniques, as they were located directly on the Cu support and not along the edges of the support (Figure S1). Similarly, dip coating methods presented additional challenges for imaging the NCA particles by TEM techniques. Directly dip coating the half-moon TEM grids into a suspension of NCA particles resulted in the assembly of relatively few particles on the grids (e.g., on average, there would be a single particle located on the edges of the TEM grid; Figure S2). An alternative method was sought to apply the particles onto the TEM grids that both increased the number of particles transferred and resulted in a distinct separation between the particles.

**Figure 1:**
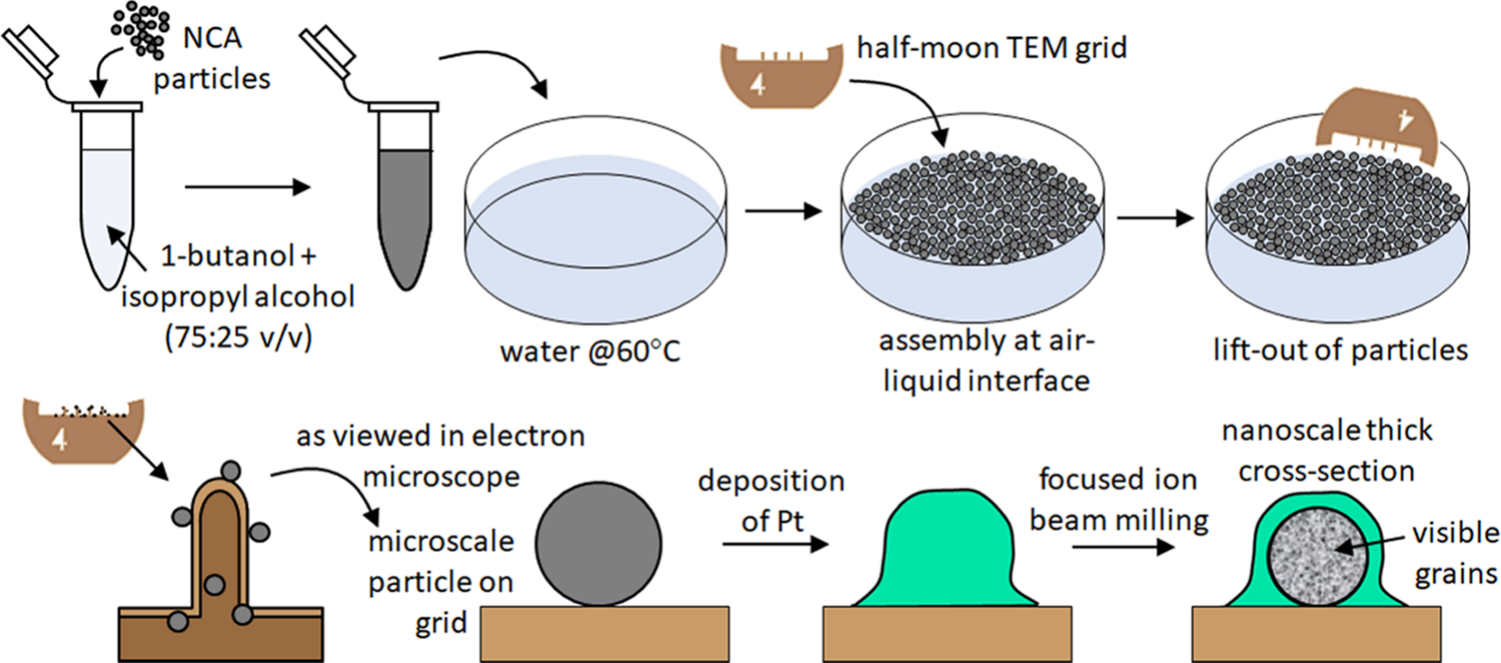
Schematic depiction of the procedures used for manipulating microscale lithium containing nickel cobalt aluminum oxide (NCA) particles by solvent-assisted dispersion, assembly at an air–liquid interface, and a subsequent transfer to a half-moon transmission electron microscopy (TEM) grid for thinning by focused ion beam (FIB) milling.

A method of concentrating the NCA particles at an air–liquid interface (i.e., forming a monolayer at the interface) followed by a transfer of these particles to a half-moon TEM grid (i.e., transfer to the TEM grid by withdrawal from beneath the assembly of particles at the air–liquid interface) was selected as an alternative approach to preparing the samples for further analysis (Figure 1). The formation of a layer of particles at an air–liquid interface followed by transferring these particles to a solid substrate has been demonstrated for creating monolayers prepared from a range of particles.^68,69^ These demonstrations have included both nanoscale^70^ and microscale particles,^71^ and these methods can even be extended to the transfer of insoluble particles.^72^ A method that we have previously found to be effective for preparing a variety of particles at an air–liquid interface is to initially prepare the particles as suspensions in a mixture of alcohols, such as 1-butanol and isopropyl alcohol, prior to the formation of an assembly of these particles at an air–liquid interface.^73,74^ It has also been reported that a higher ratio of 1-butanol can yield an improved dispersion of particles at an air–water interface due to the differences in solubility and evaporation rates between the water and 1-butanol phases.^73^ The success of this approach has been attributed to both the solubility of the alcohol in aqueous media and the influence of the 1-butanol on the surface tension of this interface.^75^ These properties each assist in the formation of particle assemblies at an air–water interface upon the addition of 1-butanol. Gently heating these aqueous dispersions prior to applying particles from an alcohol suspension results in the formation of an assembly of particles at the air–liquid interface. This approach has been utilized, for example, to create assemblies of microscale niobium pentoxide (Nb_2_O_5_) particles from suspensions in 1-butanol, followed by their transfer to a solid support through withdrawal of the support from beneath the air–liquid interface.^76^ Similar methods have been used to prepare a layer of particles as sacrificial templates against which metals are electrodeposited to create structured electrocatalysts of, for example, nickel or platinum.^74,77^ Additional solvent systems can also be utilized in this process, and understanding their interactions with the suspended materials is important to forming a successful film while also minimizing damage or degradation to the particles.^78^ This air–liquid assembly approach for the transfer of a monolayer of particles to a solid support material was adapted in this study for the direct transfer of NCA particles to half-moon TEM grids (Figure 1).

Various suspensions of NCA particles were prepared to evaluate their ability to form an assembly of these particles at the air–liquid interface. From these analyses, we selected a mixture of 1-butanol and isopropyl alcohol (75:25, v/v) as an ideal solvent system for the NCA particles. The NCA particles were prepared as suspensions in each solvent system (e.g., 1-butanol, isopropyl alcohol, 1-methanol, 1-hexanol, and mixtures therein), and subsequently these suspensions were each carefully added to an air–water interface. Care was taken to ensure that the NCA particles were added in a dropwise manner to the interface by a microliter volume pipettor such that the resulting assemblies were not significantly disturbed and to prevent the formation of larger aggregates that would settle to the bottom of the container. Various methods were attempted such as adding the alcohol-based suspension toward the edges of the container, which assisted in minimizing disruptions to the assemblies formed at the air–water interface. Of the solvent systems we evaluated, most were able to create an initial dispersion of NCA particles, but many did not form a sufficient dispersion of the NCA particles at the air–liquid interface and most resulted in the formation of aggregates of particles at this interface. Likely a lack of miscibility of some solvents (e.g., 1-hexanol) with the aqueous phase was a significant factor that influenced these outcomes. We determined that a mixture of 1-butanol and isopropyl alcohol (prepared in a volume-to-volume ratio, v/v, of 75:25) was able to form a relatively uniform dispersion of the particles at the air–liquid interface, as observed by eye, when compared to the other solvent systems evaluated in this study. And, importantly, this mixture enabled the formation of an assembly that could transfer to the half-moon grids as a dispersed coating of NCA particles. The coating contained a sufficient separation between the particles, including those particles along the edges of the grids for the ease of viewing by TEM techniques. In contrast, a suspension of particles in 1-butanol also created a dispersion of NCA particles, but the subsequent coatings that were transferred to the TEM grids contained fewer particles along the edges of the half-moon grids. It has been previously reported that differences in the solubility parameters of the water and alcohol are key factors contributing to the dispersion of the particles at the air–liquid interface.^73^ Without an appropriate difference in the solubility parameters of the solvents, the particles did not form a stable and well-dispersed assembly at the air–liquid interface. The addition of isopropyl alcohol to 1-butanol was necessary to assist in the formation of a more uniform film of NCA particles on the TEM grids, possibly due to a more uniform dispersion of the particles at the air–liquid interface and its influence on changes in the surface tension of the solvent front during evaporation. Other reports have shown that the use of low-surface-tension solvents such as isopropyl alcohol or ethanol can induce Marangoni flows that can enable the formation of more uniform films by reducing the phenomenon known as the coffee ring effect. Marangoni flow occurs when gradients of surface tension and temperature result in mass transport at the interface of the gradient.^79^ This effect occurs during solvent evaporation from a suspension of particles supported on a substrate (i.e., drying of the half-moon TEM grid after the particles have been lifted from the air–liquid assembly).^80^ By enhancing the Marangoni flows within the liquid phase, a more uniform film of particles can be achieved during solvent evaporation.^81^ Additional studies are needed to determine the effects of these solvent systems in further detail on the dispersion of the microscale particles both at the air–liquid interface and during solvent evaporation upon TEM grids. When selecting a solvent system for preparing the coatings on the TEM grids, it was important to identify a solvent system that could disperse the particles both in solution and at the air–liquid interface to control wetting of the substrate and solvent evaporation rates and possibly to influence the Marangoni flows within the solvent systems during the transfer of the assemblies to the TEM grids.

### Assessing the Potential Impacts of the Solvent Systems on Integrity of the Microscale Particles

**Figure 2:**
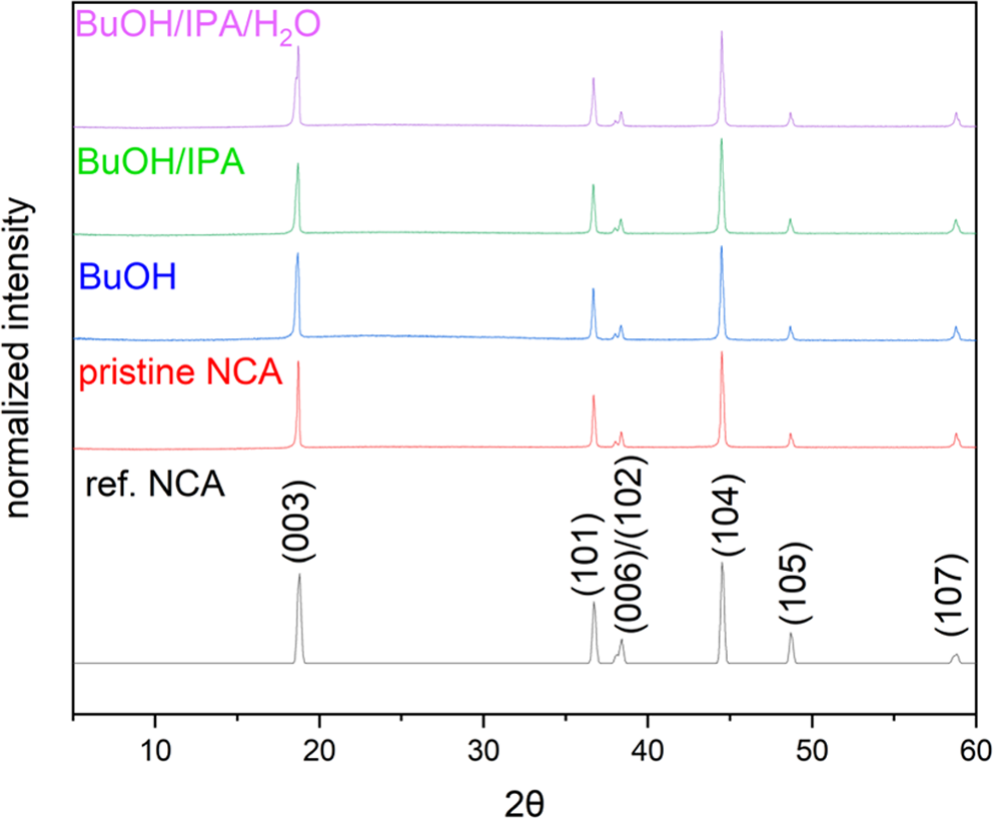
X-ray diffraction (XRD) patterns of the NCA particles: (i) as purchased, or pristine; (ii) after immersion in 1-butanol (BuOH); (iii) after immersion in a 75:25 (volume-to-volume, v/v) mixture of 1-butanol and isopropyl alcohol (IPA); and (iv) after immersion in a solution containing 1-butanol, IPA, and water (prepared in a ratio of 37.5:12.5:50, v/v/v, respectively). The diffraction patterns were each normalized to their maximum intensity at the (104) reflection. Also included is a previously reported diffraction pattern for NCA as a reference material (ICSD no. 19963).^58^

Dispersion of the NCA particles into the solvents might have a negative influence on the crystallinity and composition of the microscale particles due to potential reactivity or leaching of the microscale particles. The potential changes in particle crystallinity were evaluated using X-ray diffraction (XRD) techniques. Monitoring for structural changes as a result of exposing the particles to the solvents used to prepare the dispersions and assemblies of particles at the air–liquid interface was used as one means of assessing potential degradation of the microscale particles. For the purposes of this control study, the NCA particles were suspended in one of three different solvent systems for 3 h. These solvents selected for this study were (i) 1-butanol, (ii) a mixture of 1-butanol and isopropyl alcohol prepared in a volume-to-volume ratio (v/v) of 75:25, and (iii) a mixture of 1-butanol, isopropyl alcohol, and water prepared in a ratio of 37.5:12.5:50 (v/v/v). The period of 3 h was designed to be significantly longer than the time typically required to prepare the dispersions of particles, assemble the particles at the air–liquid interface, and transfer these assemblies to the half-moon grids (e.g., ∼30 to <60 min). After 3 h, the suspensions were each centrifuged and solvents were decanted to isolate the suspended solids. The solids were dried under vacuum overnight at room temperature to assist with removing residual solvents from the samples prior to further analysis. The crystallinity of the particles after their immersion in each solvent system was compared to that of pristine NCA particles as well as to a published reference for NCA materials (Figure 2). All diffraction peaks observed in the XRD plots were consistent with the reference NCA material (ICSD no. 19963).^58^ There were no significant changes in the peak positions, nor were there any additional peaks observed in these plots for the NCA particles after their exposure to the solvent systems. There were, however, slight changes to the relative peak intensities of some particles after solvent exposure (Table S1). In particular, after 3 h of exposure to 1-butanol, the peak intensity of the (006) planes relative to that of the (104) planes exhibited a 27% change from the peak ratio observed for the pristine sample. The same relative peak intensities exhibited a change of 10% and 9%, respectively, after suspending the particles in a mixture of 1-butanol and isopropyl alcohol (prepared at 75:25, v/v) or this same mixture when combined with water in a 50:50 ratio (v/v). Particles suspended in the mixture of 1-butanol and isopropyl alcohol also exhibited a 16% change in the relative peak intensity of the (003) and (104) planes in comparison to the pristine materials. Suspension of the particles in 1-butanol exhibited an 8% change in the relative peak intensity of the (105) and (104) planes and an 8% change in the relative peak intensities of the (107) and (104) planes. In comparison, a maximum of a 6% relative change was observed for the particles dispersed in a mixture of 1-butanol, isopropyl alcohol, and water. This latter solvent system most closely matched the solvents that were subsequently selected to prepare the assemblies at the air–water interface for transfer to the TEM grids. Cathode materials for LIBs, in particular, those that contain nickel (Ni) (e.g., NCA, LMNO, and NMC), can be sensitive to the presence of water. For example, NCA particles in the presence of water can result in leaching of lithium to a greater extent than other cathode materials.^82^ A decreased stability of NCA materials in comparison to many other types of cathode particles is likely due to the presence of aluminum, which can form LiAlO_2_ species that can readily react with water to form LiAl_2_(OH)_7_ and Li_2_Al_4_(CO_3_)(OH)_12_ species.^82−85^ There is research being performed to address the water sensitivity of Ni-rich materials such as through the application of coatings, which could also assist in decreasing production costs and improve scale-up methods by enabling the use of water-based processes during the preparation of these cathode materials for use in LIBs.^86,87^ For example, NCA particles have been coated with Li_3_PO_4_ to prevent the leaching of lithium.^88^ These prior studies also determined that a relatively thick Li_3_PO_4_ coating exhibited enhanced stability without impacting the electrochemical performance of these particles. Such coatings would also likely enhance the stability of these materials during their exposure to the solvent systems used in the assembly at an air–liquid interface and transfer to a TEM grid for processing by FIB techniques and analysis by TEM methods.

In addition to XRD analysis of the NCA particles following their exposure to different solvent systems, potential changes in the morphology, shape, and size of the particles were assessed by SEM imaging techniques. The particles soaked in the solvent systems all exhibited a similar appearance to that of the pristine particles (Figure S3). It is likely that there were minor structural changes to some of the primary NCA particles following their exposure to the solvent systems, as exhibited in the relative changes to the XRD reflections (Table S1), but these changes could not be observed by the SEM analyses. Following their assembly at and transfer from the air–liquid interface, the NCA particles did not exhibit any discernible changes to their shape, size, and morphology as observed by SEM (Figure S4). The solvent-soaked samples and those transferred from the air–liquid assemblies each contained isolated, microscale secondary particles and primary, nanoscale particles that were not observed in the pristine NCA particles. There were, however, relatively few primary particles present in any of these samples and the larger, microscale particles largely retained their size and shape after solvent exposure. Although there is some degradation of the NCA particles, such as the separation of primary particles from the secondary particles, the majority of the NCA materials remain intact when dispersed in the solvents. These minor changes to the secondary NCA particles could have resulted from leaching of Li, Co, and/or Al from the samples. These effects could be more pronounced in NCA materials in comparison to other types of cathode materials (e.g., LFP) that are less sensitive to water. The results do, however, suggest that the solvents used to prepare the assemblies for transfer from the air–liquid interface to the TEM grid did not result in significant changes to the NCA particles. Care must still be taken to minimize the duration of the exposure of the microscale particles to solvents during the processes of assembly and transfer. Likely due to the variations in their sensitivity to moisture, each type of cathode material will need to be individually assessed for the appropriate amount of time the particles can tolerate solvent exposure during these processes. Possibly, additional solvent systems will also need to be explored, but the processes of assembly and material transfer could be extended to many other materials.

### Preparation of Sample Cross Sections Using Modified Focused Ion Beam Milling Techniques

**Figure 3:**
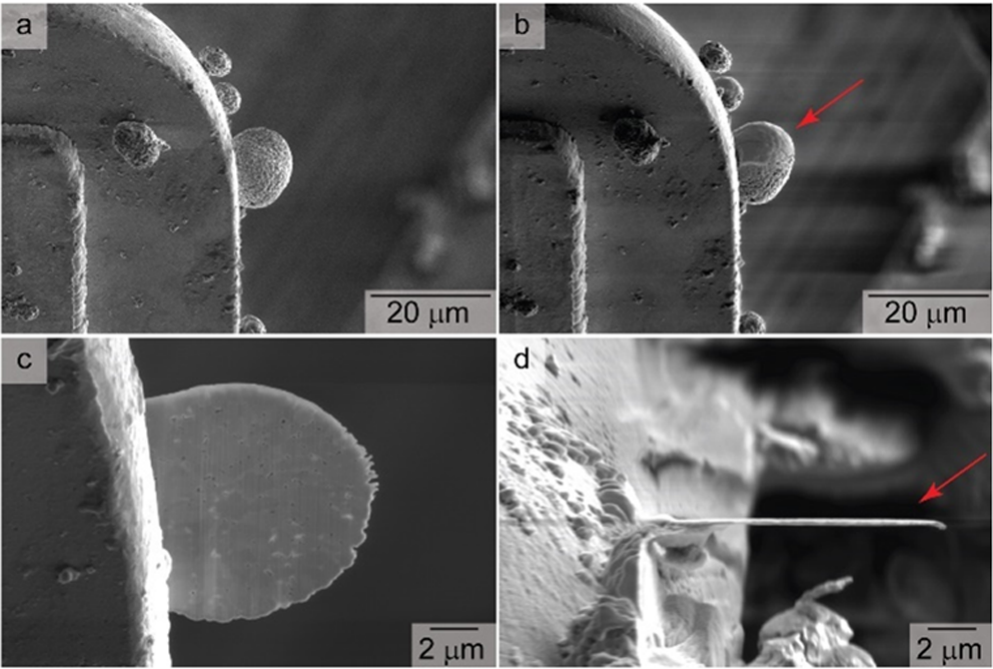
Scanning electron microscopy (SEM)-based images obtained at a tilt angle of 52° for NCA particles supported on a half-moon TEM grid during the preparation of a thin cross section for TEM analysis. These images depict (a) the pristine particles, (b) the coating of a selected particle with a protective Pt layer (as indicated by the red arrow), (c) the cross section of this particle prepared by FIB milling, and (d) the top-down view of this cross section depicting its thin profile, as indicated by the red arrow.

Following the assembly of the NCA particles at the air–liquid interface, these particles were withdrawn from solution by a selective transfer of the particles to half-moon TEM grids (Figure 1). This transfer of particles from an air–liquid interface to a solid support has been previously used to prepare thin films by assembly of many different types of particles (e.g., polymers,^77^ ceramics,^89^ and metals^70,74,76^). Assemblies of NCA particles with a sufficient spacing between the particles (i.e., did not form a densely packed layer) were sought to enable their analysis by TEM techniques. The NCA particles transferred to the half-moon TEM grids were analyzed by SEM techniques to select isolated particles for the FIB milling procedure. When a particle was selected for FIB milling, it was desirable that the selected particle had sufficient separation from neighboring particles (Figure S5). In addition, when viewing the grid by SEM analysis along the same direction as the TEM beam, those particles located along the edges of the protruding “fingers” or prongs of the TEM grid were chosen for FIB milling. These particles would be more easily visualized by TEM methods given the fact that particles located on the face of the half-moon grids would be supported on a 35 μm thick section of Cu (Figure 3a). Samples analyzed by FIB-assisted lift-out are typically placed along the edges of the prongs of the half-moon TEM grid for this reason. Two types of half-moon TEM grids were assessed for their ease of use with the transfer of NCA particles from the assemblies at the air–liquid interface to the Cu support. These grids had either two or four prongs to support the particles. The grids with four prongs (each prong with a dimension of 80 μm × 35 μm × 190 μm; *W* × *D* × *H*) were preferred due to the increased surface area along the edges of these prongs for supporting the NCA particles and, therefore, increasing the chances of finding particles with the desired spacing from neighboring particles. The grids with two prongs had less surface area along the edges of their prongs but also wider prongs (250 μm × 35 μm × 190 μm; *W* × *D* × *H*). We observed that particles assembled onto either type of Cu grid exhibited a relatively strong interaction with the grid, such that the mechanical forces of handling these grids during loading in and out of tools for imaging by both SEM and TEM, and while moving to and from the storage containers, did not dislodge these particles. The particles adhered sufficiently well to be imaged multiple times and to be reanalyzed after many months of storage.

After the particles of interest were identified for further analysis, nanoscale cross sections of the selected NCA particles were prepared by FIB milling. The first step of this process was to apply a protective layer of Pt onto the individual NCA particles to prevent damage from the incident Ga^+^ during FIB milling. Two layers of Pt were deposited on the NCA particles. The first layer was deposited with the assistance of a focused electron beam, and the second layer was deposited by use of a focused ion beam, as is the standard practice in preparing FIB cross sections of a sample. The electron beam induces less damage to the sample than the Ga^+^ beam, which is used to prepare a thin coating with Pt. A sufficiently thick layer of Pt was needed prior to FIB milling, so a thicker layer of Pt was subsequently deposited under the ion beam.^30^ Care was taken to use lower beam energies during each of these steps to minimize damage to the NCA particles during the Pt deposition.^30^ Platinum with an overall thickness of ∼100 nm was used to protect the surfaces of the microscale particles from the Ga^+^ beam during the milling process (Figure 3b). Thicker layers of Pt can be deposited when desired to further protect the samples from local changes in crystallinity at the surfaces of the materials and to avoid embedding Ga^+^ within the sample.

Following Pt deposition, the Pt-capped NCA particles were selectively thinned using a Ga^+^ beam. The central region of each cross section was selected, and the particle was milled on either side. These cross sections were created with a thickness of <100 nm (e.g., Figure 3c,d) to enable the transmission of electrons during their characterization by TEM techniques. Compared to the standard procedures for FIB-assisted lift-out of nanoscale sections of a sample, our modified technique significantly reduced the time required to prepare nanoscale cross sections of NCA particles. Using the outlined procedures, cross sections were typically prepared in <1.5 h, which included the time to locate a particle of interest, to prepare a protective Pt layer, and to perform the FIB milling to isolate the desired cross section of the particle. Most of the variation in time required to prepare each sample was due to variations in the length of time required to deposit a protective Pt layer. Larger particles required more time for Pt deposition, whereas smaller particles took less time. Our experience with performing FIB-assisted lift-out on these and similar materials is that a significantly longer time (e.g., >3×) was required to isolate individual cross sections. The FIB-assisted lift-out of semiconductor materials (e.g., silicon-based samples), for which the FIB-assisted lift-out workflow was created, can take 5 h based on literature reports.^35^ Our experience is that the success and duration of a FIB-assisted lift-out procedure is not only correlated with the skill of the worker but also relies on the mechanical and electrostatic properties of the sample. By transferring the microscale particles onto the half-moon grids prior to the creation of their nanoscale cross sections, the methods introduced herein avoid these challenges and enable more particles to be analyzed on a single half-moon grid. We estimate an increase in the number of samples per TEM grid of at least 5× from that achieved using a FIB-assisted lift-out process based on our experience. This alternative method for preparing nanoscale cross sections of microscale particles has a higher sample throughput and offers a process that is accessible to more users (e.g., avoiding the need for the training and skill development involved in sample lift-out as performed by FIB-assisted techniques). Furthermore, FIB-assisted lift-out techniques typically cannot be scaled up to analyze a statistically relevant number of samples due to the time and skill required to prepare individual cross sections of the sample for analysis. Thus, characterization is often based on the few particles successfully prepared by FIB-assisted lift-out procedures. The direct-support workflow established in this work enables a high-throughput process for high-resolution single-particle analysis that can be used to obtain statistically relevant data from a number of single particles. In addition, these procedures can assist in the development of a variety of microparticles, including those used in LIBs, zinc-based batteries,^90^ and other industrial applications. Future studies will be used to prepare thin cross sections of microscale particles of a variety of compositions; of particular interest is the use of coatings on cathode materials for enhanced durability. The uniformity of these coatings can have implications for their long-term cycling capabilities in a LIB, and hence assessing the effectiveness of the coating procedures (i.e., through characterization methods) is essential. For example, coatings have shown promise in preventing degradation of NCA materials when exposed to moisture, but the composition of the coating material relative to the inner can often be a challenge.

### Analysis of the Cross Sections of Microscale Particles by TEM Techniques

**Figure 4:**
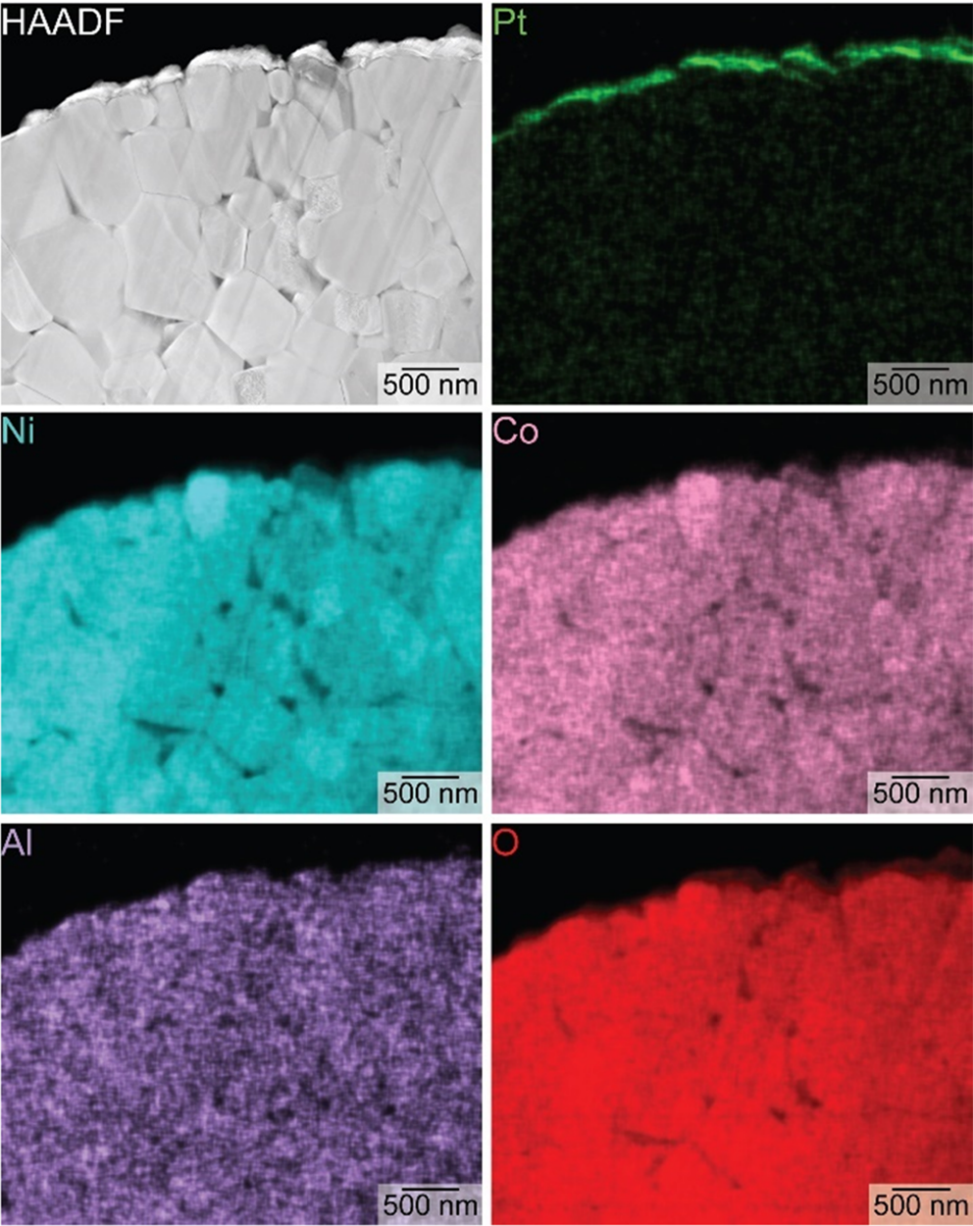
Transmission electron microscopy (TEM)-based analyses of a nanoscale, thin cross section of a microscale NCA particle. A high-angle annular dark-field (HAADF) image depicts individual grains within the NCA particle. These grains correspond to variations in the elemental content (Ni, Co, Al, and O) throughout the particle as observed in the elemental maps as obtained by energy-dispersive X-ray spectroscopy (EDS). The Pt layer on the NCA particle served as a protective layer during FIB milling.

After the FIB milling procedure, the half-moon TEM grids were stored in a FIB sample holder until TEM analysis. These grids were loaded into the TEM system by using a standard TEM holder for analysis of the thinned, electron transparent cross sections. Samples to be imaged by TEM techniques must typically be ≤100 nm thick, but the exact limit to this thickness can vary depending on the accelerating voltage of the microscope.^23^ Cross sections of the NCA particles prepared using the FIB milling procedure outlined herein were sufficiently thin for TEM analysis (Figures S6 and S7). Elemental composition of the samples was assessed using energy-dispersive X-ray spectroscopy (EDS) techniques. As expected, the samples were found to contain Ni, Co, Al, and O, in agreement with the components of NCA (Figure 4). The electron microscope used in this study was not capable of detecting X-rays produced by Li; hence, these species were not observed in the EDS results. Additional species were also observed in the spectrum obtained by EDS analyses (e.g., Figure S8). Some of the species were localized to the outer regions of the sample, such as Pt and Ga. These species corresponded to the Pt protective layer, which received the highest dose of Ga^+^ in the preparation of the cross sections by FIB milling. In addition, a Cu signal in the spectrum was also observed due to contributions from the Cu half-moon TEM grids. The relative Ni, Co, and Al contents in these particles—that had been dispersed in solution, assembled at the air–water interface, transferred to the Cu grids, and processed by particle-selective FIB milling—were compared to the relative amounts of each element present in the original sample. The ratios of Ni to Co to Al, as determined by comparing the relative areas of the associated Kα peaks, were 8.71 ± 0.03:1.58 ± 0.01:0.35 ± 0.02. These ratios closely matched the expected values as reported by the manufacturer (Ni/Co/Al = 8.15:1.5:0.35). These results suggest that the primary content of the NCA particles was preserved throughout the process. This technique could be extended to analyzing the elemental composition of other microscale particles in future studies such as particles where the composition of its materials is unknown.

### Analysis of Irregularly Shaped Particles

The preparation of nanoscale cross sections of battery cathode materials for particle assembly and FIB milling on a half-moon grid were extended to irregularly shaped particles of LMNO. These LMNO particles were also secondary microscale particles composed of primary particles that had nanoscale dimensions. The overall size and shape of the secondary particles were, however, less regular than those of the NCA particles (Figure S9). The LMNO particles had a larger size distribution and relatively inconsistent shapes (e.g., nonspherical) in comparison to the NCA cathode materials. The methods outlined here were used to assemble the LMNO particles at an air–liquid interface and to transfer these materials to half-moon TEM grids. The FIB preparation of cross sections and analysis of these particles by TEM techniques presented some additional challenges. The LMNO particles exhibited a less uniform packing of the primary particles within the secondary particles, which resulted in the formation of relatively large voids in comparison to the NCA particles. Of note, the extent of these large voids in the secondary particles was only determined after the FIB milling process to create the nanoscale cross sections.

The void spaces within the LMNO particles presented challenges when preparing and analyzing thin cross sections. The extent of the voids within these materials was not well understood prior to selection of a particle for thinning. Variations in the true thickness of the LMNO particles resulted in a variable rate of FIB milling across these irregular surfaces. After assembly and transfer of the particles to a half-moon TEM grid, a Pt protective layer was deposited over the surfaces of selected particles prior to creating cross sections by FIB milling. This protective coating had to be thicker than that used in preparing cross sections of the NCA particles. For example, an ∼700 nm thick layer of Pt was used for protecting the LMNO particles versus ∼100 nm thick Pt on the NCA particles. This increased thickness was necessary, in part, to ensure that the LMNO particles remained attached to the half-moon TEM grid. Even with the thicker Pt protective layer, the voids present within the particle often resulted in a relatively minimal amount of sample remaining for further analysis. In addition, achieving a cross section with a uniform thickness was also challenging due to the nonuniform milling rate across the sample. Extensive FIB milling could result in a loss of structural integrity within the sample and/or a decreased adhesion to the grid. These challenges extended into the analysis of the samples by TEM. Variations in the thickness of the resulting cross sections arising from the porosity and the interior texture of the sample made it difficult to discern the overall structure of the secondary particle (Figure S10). Additionally, the presence of voids within some regions of the sample resulted in more care being needed when analyzing the sample under the focused electron beam of the TEM, such as lowering the electron dose to avoid damaging the microscope camera. Supporting the samples on the half-moon TEM grids not only provided a relatively high contrast during TEM analysis in comparison to the contrast achievable using standard TEM grids (e.g., Formvar/carbon-coated copper mesh grids) but also decreased the stability of the materials under the electron beam. Given the irregular thickness of the sample and the need to avoid high electron fluxes on the camera, relatively small regions of the sample were analyzed in contrast to those studied for the NCA particles. There were also challenges associated with the EDS analysis of the cross sections of the LMNO particles due to the thicker protective layer of Pt (Figure S10). The dominant EDS signals were from Pt within the protective layer and Cu from the TEM grid (Figure S11). Elemental analysis of the secondary particles did confirm the presence of Mn, Ni, and O (Figures S10 and S11). Further, a Ga signal, due to the ion beam, was found to be localized to the Pt protective region (Figure S10). Obtaining a clear image of the structure of the secondary particles in this sample required more replicates than analyzing the NCA particles due to the presence of the voids within the secondary LMNO particles. The presence of the voids increased the processing time for these samples when creating nanoscale cross sections. The techniques demonstrated were more ideal for use with secondary particles that are assembled from densely packed primary particles, but this method still enables the analysis of irregular particles such as the LMNO particles that would otherwise be even more challenging to study on an individual particle basis when FIB-assisted lift-out techniques.

## Conclusions

Nanoscale cross sections of microscale particles can be prepared using a custom focused ion beam (FIB) milling procedure that enabled a detailed, high-resolution analysis of these materials by transmission electron microscopy (TEM). This workflow was performed using cathode materials for lithium-ion batteries such as lithium containing nickel cobalt aluminum oxide (NCA) particles. These cathode particles were suspended in a mixture of 1-butanol and isopropyl alcohol (75:25, v/v), drop-cast onto water to assemble a layer of particles at the air–liquid interface, and transferred to a half-moon TEM grid by submerging and withdrawing this substrate from beneath the air–liquid assembly. Collection of the particles at the air–liquid interface enabled a higher concentration of particles to be transferred (i.e., compared to dip coating methods) while also achieving a distinct separation between single particles (i.e., compared to drop-casting methods). A relatively uniform dispersion of particles was sought for both the ease of selecting individual particles for further analysis and ensuring that a high-resolution characterization of a cross section of a single particle would not have interference from other particles in the sample.

The air–liquid assembly process avoided the lengthy and technically challenging process of FIB-assisted lift-out, but the transfer to the half-moon grids presented its own potential challenges. Exposure of the cathode materials to solvent systems might introduce structural changes. Potential changes to these materials were assessed by X-ray diffraction (XRD) and scanning electron microscopy (SEM). A prolonged test for solvent exposure was performed by suspending the cathode particles in relevant solvent systems for 3 h, in contrast to a typical exposure to solvents of ∼30 min during the processes of particle dispersion, assembly, and transfer to the TEM grids. Under relevant conditions for the transfer from the assemblies at the air–liquid interfaces, relatively minimal changes to the structure of the NCA particles were observed by XRD and some primary particles became isolated from the secondary particles as observed by the SEM analyses. The majority of the secondary NCA particles remained intact and retained their original features. It is possible that minor structural changes resulted from the water sensitivity of these NCA particles. To circumvent this sensitivity, particles with a protective coating (e.g., Li_3_PO_4_) could improve the electrochemical durability of high Ni-content cathode materials and their stability during the air–liquid assembly.

After transfer of the particles to the half-moon grids via the air–liquid assembly, nanoscale cross sections of these particles were prepared using a dual-beam FIB-SEM system. Particles supported along the edges of the prongs of half-moon TEM grids were selected for further analysis by TEM techniques. To these particles, a protective platinum (Pt) layer was applied to minimize ion beam damage during subsequent FIB milling. The selected particles were thinned by FIB milling to prepare cross sections of sufficient thinness to achieve electron transparency. Compared to conventional FIB-assisted lift-out procedures, the methods demonstrated in this study provided a higher yield and a 5× faster throughput for the preparation of nanoscale cross sections of microscale particles (e.g., ∼1.5 vs >5 h). The developed workflow using semispherical, microscale NCA particles was extended to the analysis of more irregularly shaped lithium manganese nickel oxide (LMNO) particles. These particles were also easily assembled at the air–liquid interface and transferred to the half-moon grids but presented further challenges during the FIB milling due to their irregular texture and internal porosity. Cross sections of both the NCA and LMNO particles were prepared in a relatively short period of time, and their elemental composition and internal texture were assessed using TEM and energy-dispersive X-ray spectroscopy techniques.

This work provided a relatively high-throughput and technically less challenging method of preparing nanoscale cross sections of single microscale cathode particles through direct particle support in contrast to FIB-assisted lift-out techniques. The FIB workflow discussed in this work can be used to selectively analyze regions of the sample, which could further be extended to postmortem analysis for assessment of the stability of the coating materials. The higher-throughput analysis workflow developed herein can be used to assess, with statistical relevance, the end-of-life particles from the particles recovered from LIB coin-cells. Future studies may include tuning the selection of the solvent system to tune the particle packing efficiency at the air–liquid interface. Particles with different compositions may require alternative solvent systems to prepare an appropriate assembly for this transfer process due to variations in the properties of the particles (e.g., composition, surface chemistry, porosity, and moisture sensitivity). When the solvent system is tuned, it is recommended to perform XRD analyses of the particle (e.g., other battery materials) as a function of exposure to the solvent system to assess potential changes to the phase and composition of its crystalline structure. Studies using materials that are particularly sensitive to the ion beam may require lower beam energies or alternative types of ions (e.g., Xe, He) to minimize the potential for FIB-induced damage, and alternatively an analysis using ultramicrotome techniques may be preferable, although a correlative analysis of single microscale particles would be more challenging. Furthermore, compared to FIB lift-out techniques, where statistical analysis of many particles is also challenging due to sample preparation time and the difficulties associated with material handling, the demonstrated FIB workflow could be used for a high-throughput analysis of a variety of materials. The direct support on a TEM grid used in this modified FIB workflow could be extended to enable high-resolution, nanoscale analysis by TEM techniques of other types of microscale particles, including core–shell materials, Janus particles, microplastics, and other types of materials prepared by self-assembly of nanoparticles.

## Experimental Section

### Transfer of Microscale Particles to Half-Moon TEM Grids

Lithium containing nickel cobalt aluminum oxide (NCA) materials were purchased from MTI Corporation (LiNiCoAlO_2_, item no. Lib-LNCA810, MTI Corporation). These particles were suspended with the assistance of ultrasonication for 5 min in a mixture of 1-butanol [CH_3_(CH_2_)_2_CH_2_OH, 99.4%, CAS no. 71–36–3, Caledon Laboratory Chemicals] and isopropyl alcohol [(CH_3_)_2_CHOH, >99.5%, CAS no. 67–63–0, Sigma-Aldrich] prepared as a solution in a volume-to-volume (v/v) ratio of 75:25. The mixture of alcohols was prepared with NCA particles added at a concentration of 10 mg/mL. A glass Petri dish containing deionized water (18.2 MΩ·cm) was heated to 60 °C, and a suspension of NCA particles in a mixture of 1-butanol and isopropyl alcohol was added in a dropwise manner (∼7 μL per drop at an approximate rate of 1 drop per 30 s) to the air–water interface until forming a uniform layer of NCA particles at this interface.

Copper-based half-moon grids for use in transmission electron microscopy (TEM) were used as a support for the NCA particles. These TEM grids are typically used to support samples prepared by focused ion beam (FIB)-assisted lift-out (PELCO FIB Lift-Out TEM Grids containing 4 narrow posts, product no. 10GC04, Ted Pella). These half-moon grids were coated with a layer of NCA particles by dipping their posts beneath the liquid and lifting the grid through the layer of NCA particles assembled at the air–liquid interface. The copper (Cu) posts of the TEM grids served as a support for the NCA particles. These particles adhered to the TEM grids through adhesive forces that formed upon solvent evaporation. The grids supporting the NCA particles were dried in a vacuum desiccator overnight at approximately −950 mbar, and the adhered particles were thinned for TEM analysis by using FIB techniques as described below.

To characterize the potential negative influences of the solvent systems on the NCA particles, particles were transferred from the air–liquid interface to sections of polished silicon (Si) wafer following the same procedures outlined when using half-moon TEM grids as the substrate. The isolated particles were imaged by SEM techniques to assess their degradation following suspension in different solvents. Additional samples were prepared by suspending the particles directly in a series of solvents to evaluate the ability to prepare layers of particles for analysis by drop-casting and dip coating methods. Samples were prepared by directly drop-casting from these solvents onto the TEM grids and polished Si wafers. Dip coating was used to prepare samples by vertically approaching the fingers of the half-moon TEM grid to dip the grid perpendicular to the interface of the particle suspension, followed by vertical removal of the grid and subsequent vacuum drying. Lithium manganese nickel oxide (LMNO) materials provided by Nano One Materials Corp. (Batch No. S18–142A-F2–1) were also assembled on half-moon TEM grids using the same procedures as those outlined above in place of the NCA particles.

### Preparing Nanoscale Cross Sections of Selected, Individual Particles by FIB Milling

Cross sections of the cathode particles were prepared by using FIB milling techniques that were performed on a dual-beam scanning electron microscope (SEM) and FIB system. Each of the half-moon TEM grids coated with the cathode particles was held in a FIB grid holder (Ted Pella, PELCO Small FIB Grid Holder with a 12.7 mm diameter pin, product no. 15464) during sample analysis in the dual-beam system. The system used for these studies was an FEI Helios SEM NanoLab 650 SEM/FIB. Isolated particles supported on the grids were selected for thinning by FIB milling to create nanoscale cross sections for analysis by TEM. For these analyses, individual particles had to be located along the edges of the prongs of the half-moon TEM grid; otherwise, the thickness of the grid would interfere with the TEM imaging during characterization after the thinning procedure. To protect the particle of interest from damage by the ion beam processing, a thin layer of platinum (Pt) was deposited first by the electron beam at 5 kV and with a current of 0.80 nA, and subsequently a thicker layer of Pt was deposited using the ion beam at 30 kV and with a current of 0.79 nA. The Pt-protected particle was subsequently thinned using the Ga^+^ beam (Tomahawk ion column). Bulk thinning of material on either side of the particle (e.g., to analyze a cross section of the middle of a microscale particle) was performed by FIB milling at 30 kV and with a current of 2.5 nA. After preparation of an initial cross section of the sample, the dimensions of this section of sample were further thinned using an ion beam with a current of 0.40 nA. The final thickness of the sample was ∼100 nm.

### Imaging of Thinned Samples by TEM Techniques

After FIB milling of the particles, the half-moon TEM grids supporting the thinned cross sections of the microscale particles were inserted into an S/TEM (FEI Osiris X-FEG 8 scanning/TEM) for further analysis. A focused electron beam with a 200 kV accelerating voltage was used to image the samples. Energy-dispersive X-ray spectroscopy (EDS) was used to analyze the elemental composition of the samples. The EDS analyses were performed by using a Super-X EDS system with ChemiSTEM technology that integrated the signal from four spectrometers. The signal obtained during these measurements from Cu was due to the proximity of the Cu-based half-moon TEM grid, and the Pt signal was due to the protective layer deposited prior to the FIB milling procedure. A series of EDS maps were prepared depicting the distribution of elements found within the particles, which were associated with the composition identified from a survey scan obtained over an energy range of 0–10 kV.

### Analysis of NCA Particles by XRD

To ensure that the NCA particles experienced minimal structural changes due to exposure to the solvent systems used to prepare the particles at an air–liquid interface, the particles were evaluated using X-ray diffraction (XRD) techniques. The NCA particles were suspended in three different solvent systems to assess the potential impacts of typical solvents used in these preparations: (i) 1-butanol, (ii) a mixture of 1-butanol and isopropyl alcohol prepared in a 75:25 ratio (volume-to-volume, v/v), and (iii) a mixture of 1-butanol, isopropyl alcohol, and water in a ratio of 37.5:12.5:50 (v/v/v). After dispersion of the NCA particles in a particular solvent system for a total of 3 h, the suspension was centrifuged at 4000 rpm for 2 min and the solvents were removed by decanting from the isolated solids. The collected NCA particles were dried overnight in a vacuum desiccator to remove any residual solvent. X-ray crystallography data was acquired for the particles isolated from all three solvent systems, and the results are compared to those from pristine NCA particles.

The crystallography data was acquired using a Rigaku MiniFlex 6G system equipped with a 600 W Cu X-ray source operated at 40 kV and 15 mA. Each NCA sample was supported on a separate glass slide, using ∼20 mg of sample for the XRD analysis. The XRD analyses were performed over a 2θ range from 5 to 90° with a step size of 0.01° and a scan speed of 5°/min. In addition, during the XRD measurements, potential sample fluorescence was suppressed by utilizing settings to reduce X-ray fluorescence (XRF) from the sample. Results from the XRD analyses (Figure 2) were normalized by dividing all of the peak intensities by the maximum diffraction intensity [i.e., that obtained for the (104) planes].

## Supporting Information Available

Additional data obtained by scanning electron microscopy of the pristine particles; particles after suspension in different solvents; particles after assembly onto half-moon TEM grids; and additional results from elemental analyses and crystallography-based measurements (PDF)
