# Supplementary material for: Atomic-Scale Characterization of Microscale Battery Particles Enabled by a High-Throughput Focused Ion Beam Milling Technique
Source: ACS Omega. 2024 Apr 6;9(15):17467–80. doi: 10.1021/acsomega.4c00318 (PMC11025079; doi:10.1021/acsomega.4c00318)
Supplement: Supplementary file 1 — ao4c00318_si_001.pdf [file ao4c00318_si_001.pdf]

Supporting Information

Atomic-Scale Characterization of Microscale Battery Particles Enabled by a High Throughput Focused Ion Beam Milling Technique

Alexi L. Pauls, [a] *Melissa J. Radford,* [a] *Audrey K. Taylor,* [a,#] *and Byron D. Gates* [a,*]

[a] Department of Chemistry, Simon Fraser University, 8888 University Drive, Burnaby, BC V5A 1S6 (Canada)

Present Address:

[#] National Renewable Energy Laboratory, MCCS, 15013 Denver Pkwy Golden, CO, US 80401-3393 (USA)

*Email Address: bgates@sfu.ca

This research was supported in part by the Natural Sciences and Engineering Research Council of Canada (NSERC; Grant No. RGPIN-2020-06522), a Simon Fraser University (SFU) Graduate Fellowship (Alexi Pauls), and CMC Microsystems (MNT Grant No. 8651). The lithium manganese nickel oxide (LMNO) was graciously provided by Nano One Materials Corp. This work made use of the 4D LABS and the Center for Soft Materials shared facilities at SFU supported by the Canada Foundation for Innovation (CFI), British Columbia Knowledge Development Fund (BCKDF), Western Economic Diversification Canada, and SFU. Additional support was provided by 4D LABS staff member, Dr. Michael Wang, for use of the XRD. Further, A. L. Pauls would like to thank Kelsey L. Duncan and Nolan R. C. Parker for insightful discussions throughout the project.

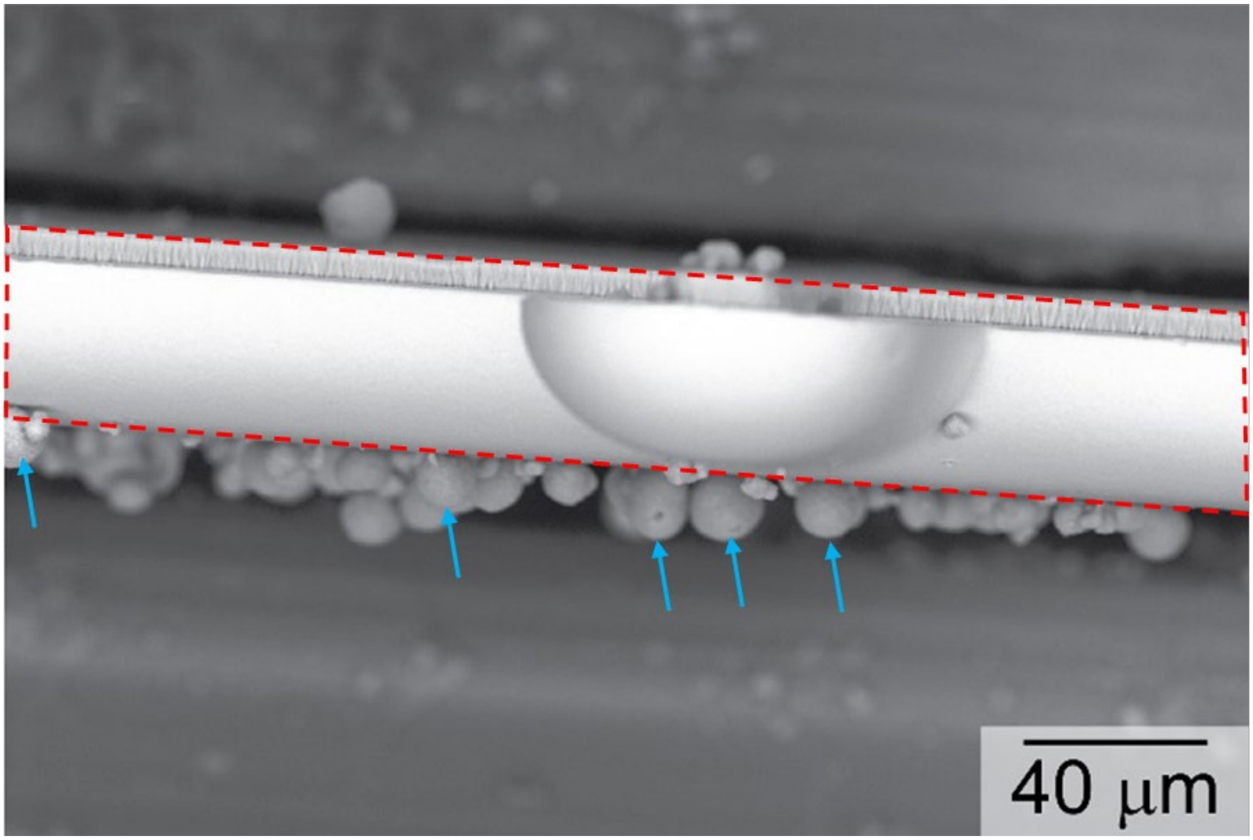

Figure S1. Top-down representative scanning electron microscopy (SEM) images of lithium nickel cobalt aluminum oxide (NCA) particles that were drop-cast onto a transmission electron microscopy (TEM) half-moon grid from a suspension in methanol. This top-down view of a prong or finger on the TEM grid (red dashed outline) depicts the lack of particles on the protruding prong, while particles formed piles upon the relatively thick regions of the grid itself (blue arrows). The collection of particles on the face of the grid extended past the depth of field where the top of the grid would still remain in-focus (i.e., are further away from the top-most edges of the TEM grid), hence only a few of the particles that are in-focus have been depicted with blue arrows. Alternative methods were sought to achieve a more uniform coating of particles upon the prongs of the TEM grids.

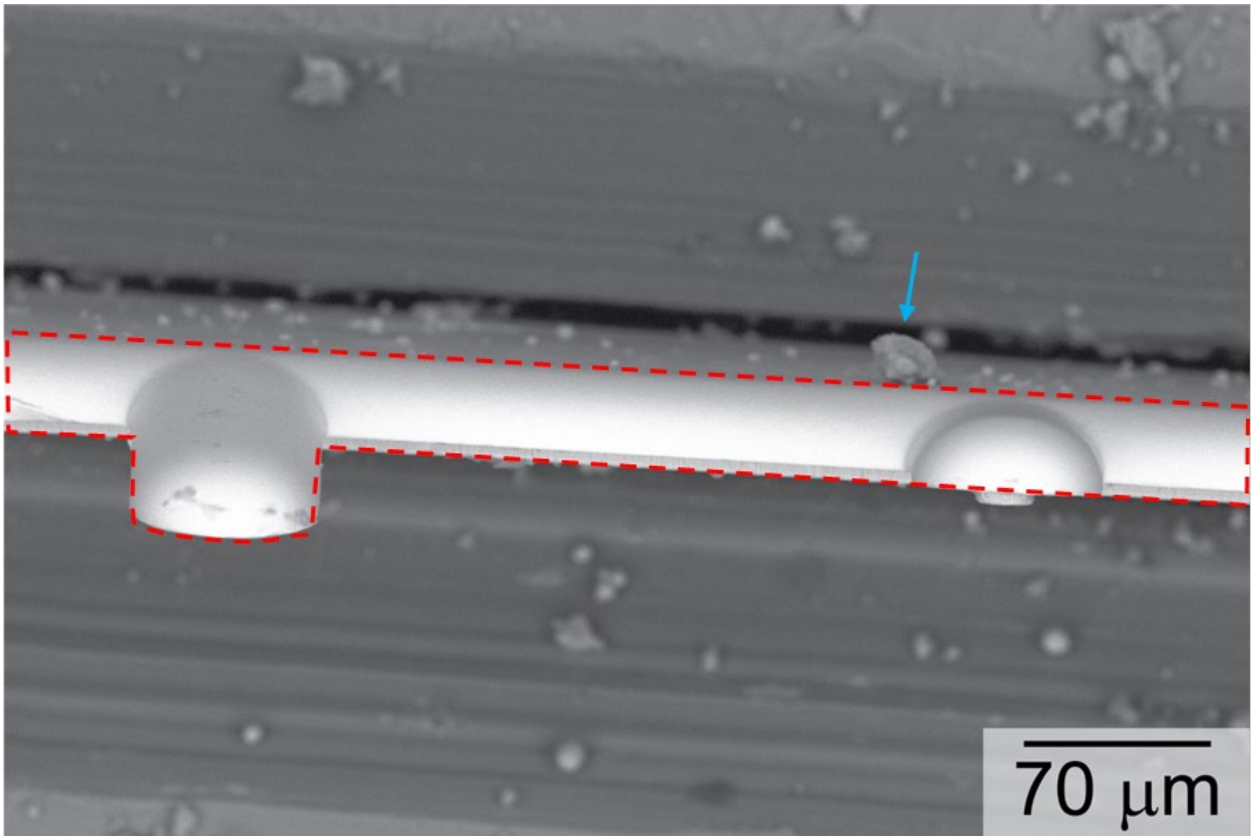

Figure S2. A top-down representative SEM image of a portion of a TEM half-moon grid that was dip coated into a suspension of NCA particles in isopropyl alcohol. The top-down view of the grid with the regions of interest have been outlined in a red dashed line. This process resulted in a lack of particles on the protruding fingers of the TEM grid, which were sought for FIB milling and subsequent TEM analysis of cross-sections of these particles. Compared to drop-casting methods (Figure S1), dip coating resulted in significantly fewer particles on the face of the grid, where only a few select particles were visible on the grid. A single particle on the face of the grid has been depicted with a blue arrow.

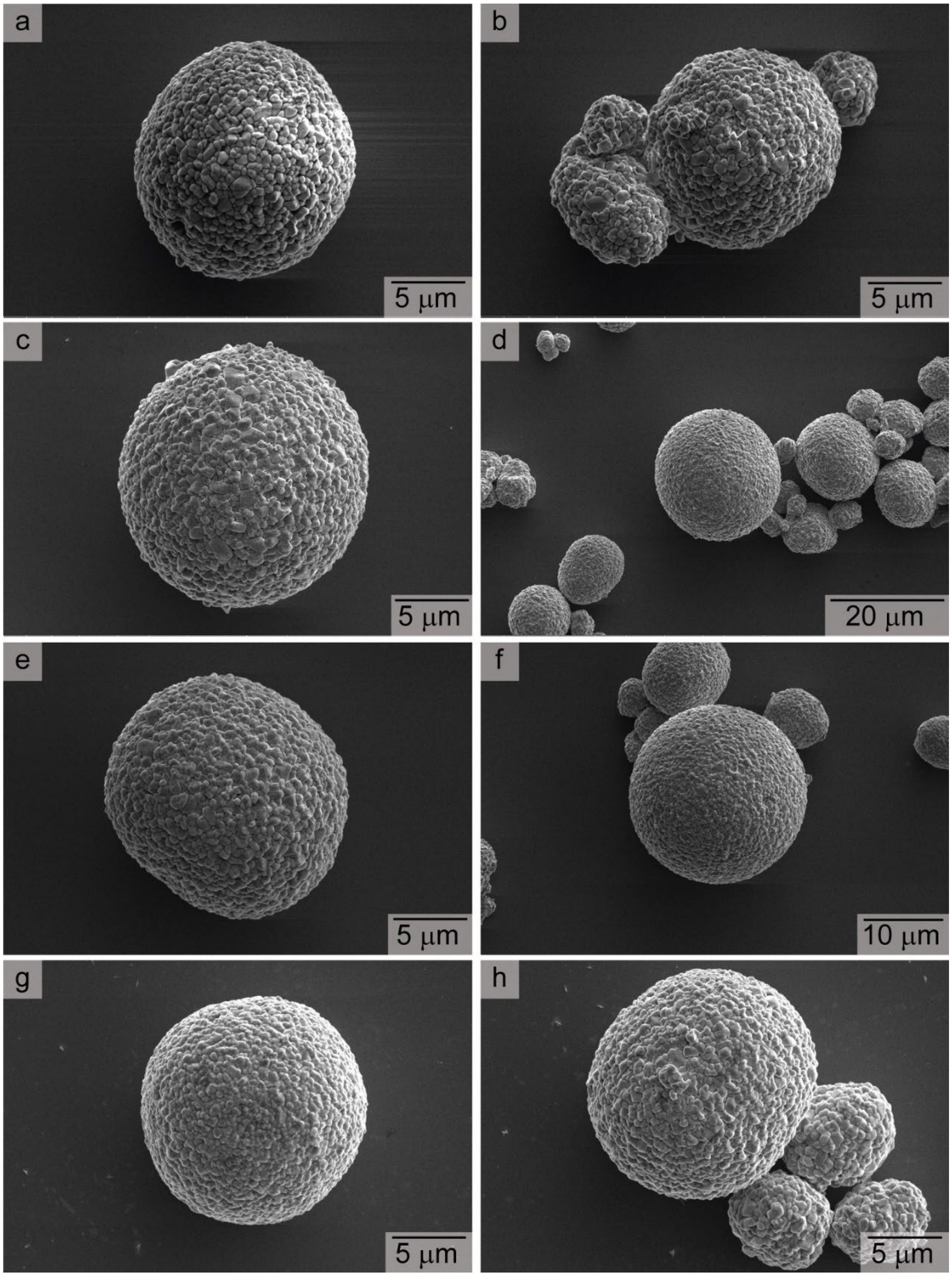

Figure S3. (a,b) Representative SEM images of lithium containing NCA particles before exposure to solvents. And SEM images of particles after immersion for 3 h in (c,d) 1-butanol, (e,f) a mixture of 1-Butanol and isopropyl alcohol (75:25, v/v), and (g,h) a mixture of 1-butanol, isopropyl alcohol, and water (37.5:12.5:50, v/v/v).

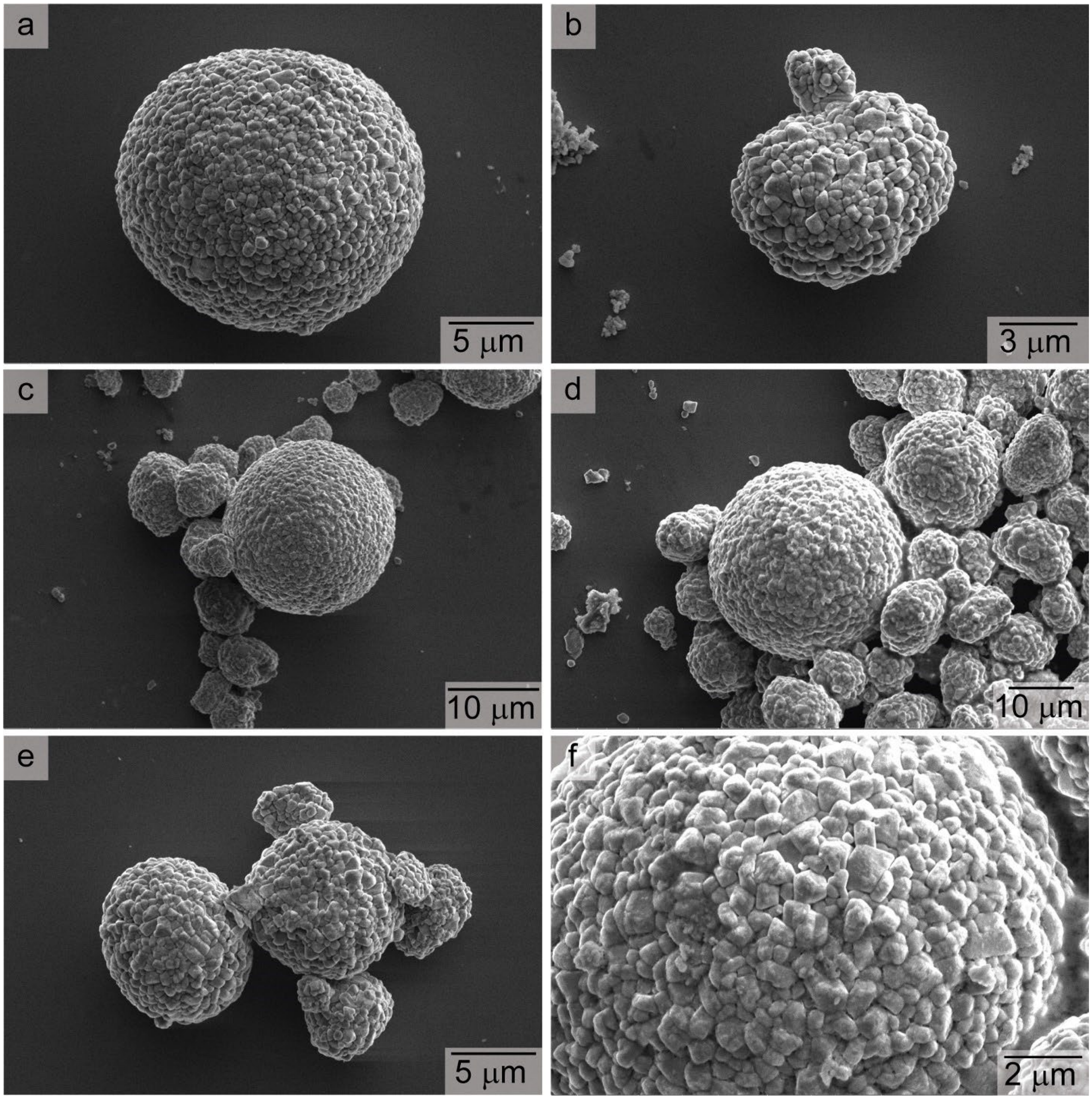

Figure S4. Representative SEM images of NCA particles after their assembly and lift-out from an air-liquid interface. (a-f) A variety of representative particles demonstrate the effects of solvents on the NCA particles. The particles were applied to an air-water interface from a suspension of particles in a mixture 1-butanol and isopropyl alcohol (75:25, v/v).

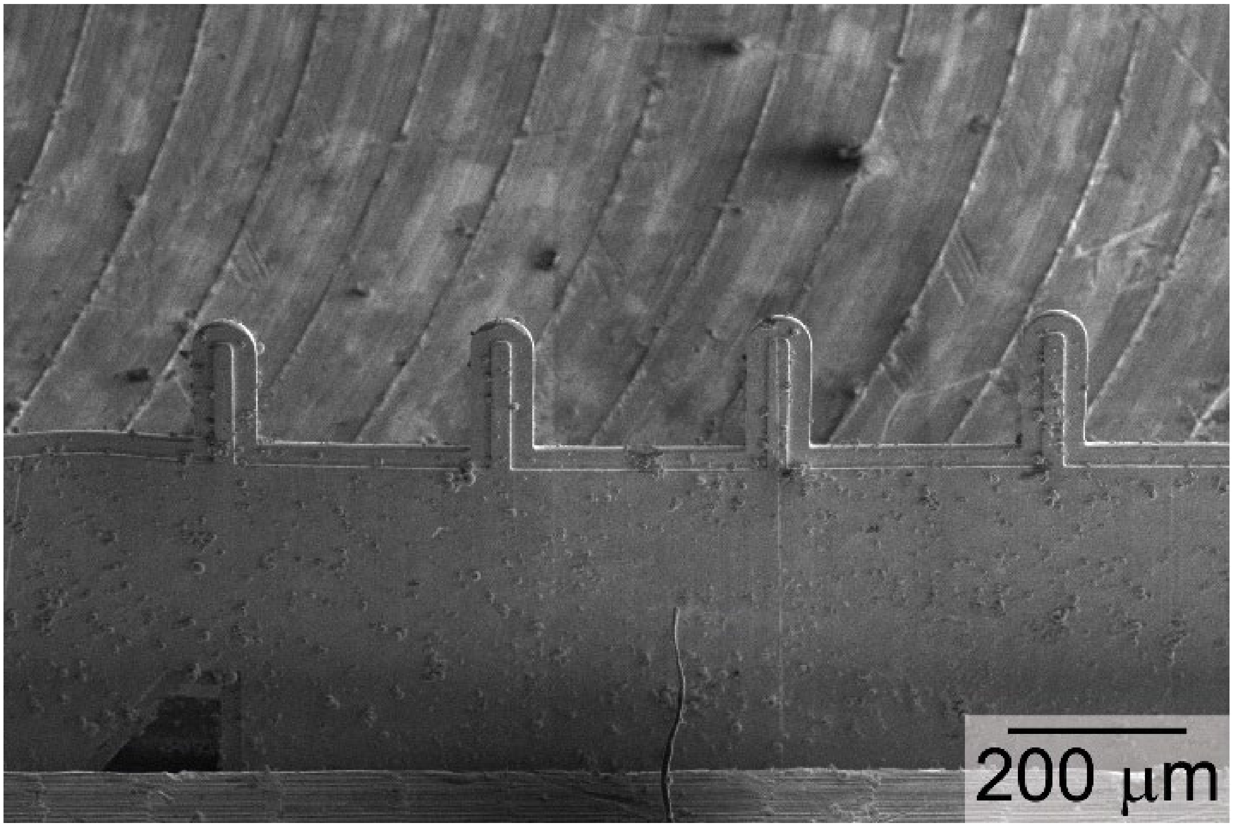

Figure S5. A low-magnification SEM image of a representative half-moon grid after transfer of lithium containing NCA particles to this TEM grid from an assembly of particles prepared at an air-liquid interface.

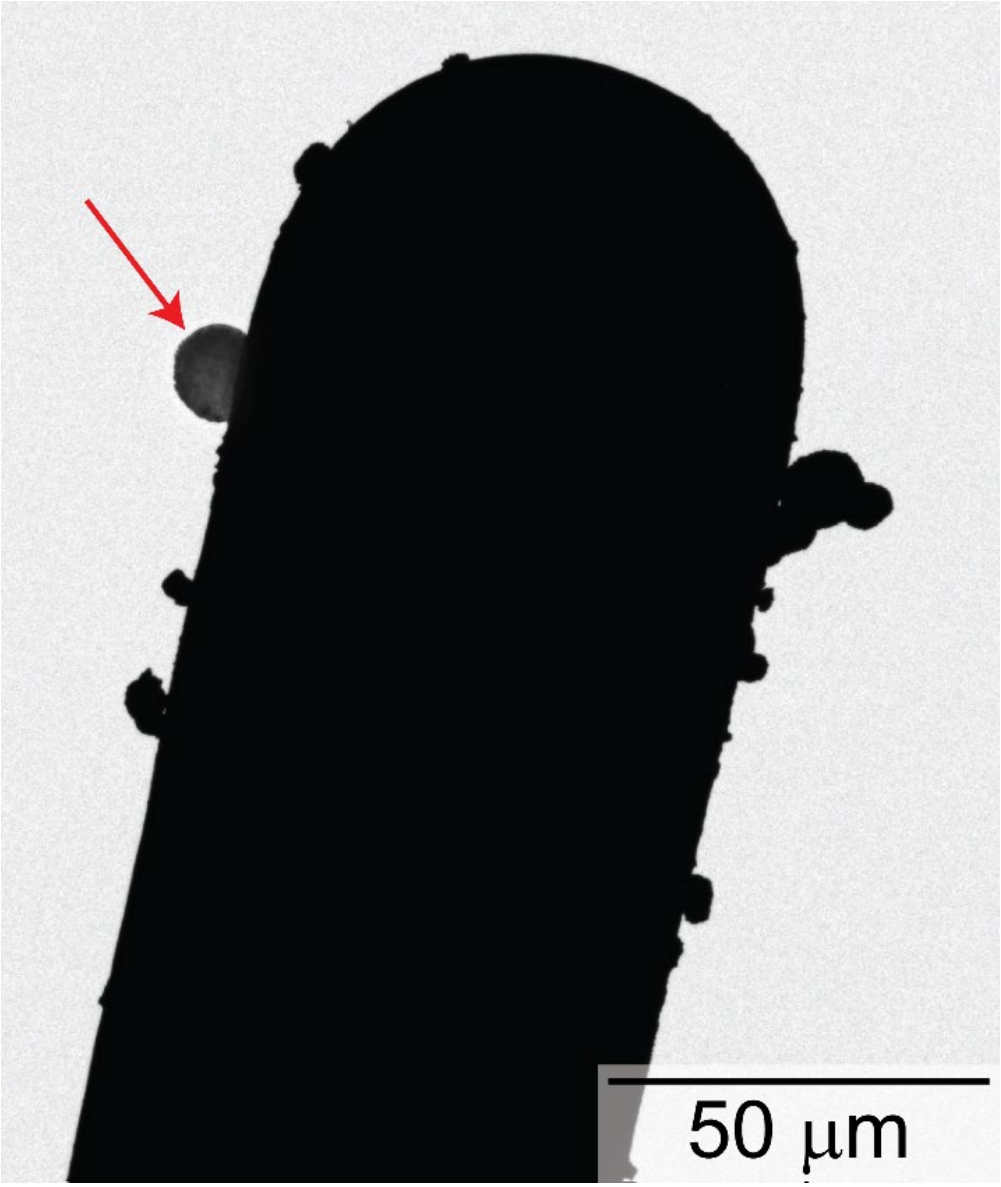

Figure S6. A low-magnification TEM image of NCA particles supported on a finger or prong of a copper (Cu) half-moon TEM grid. The red arrow points to a thin cross-section of an NCA particle as prepared by the focused ion beam (FIB) techniques outlined in the manuscript.

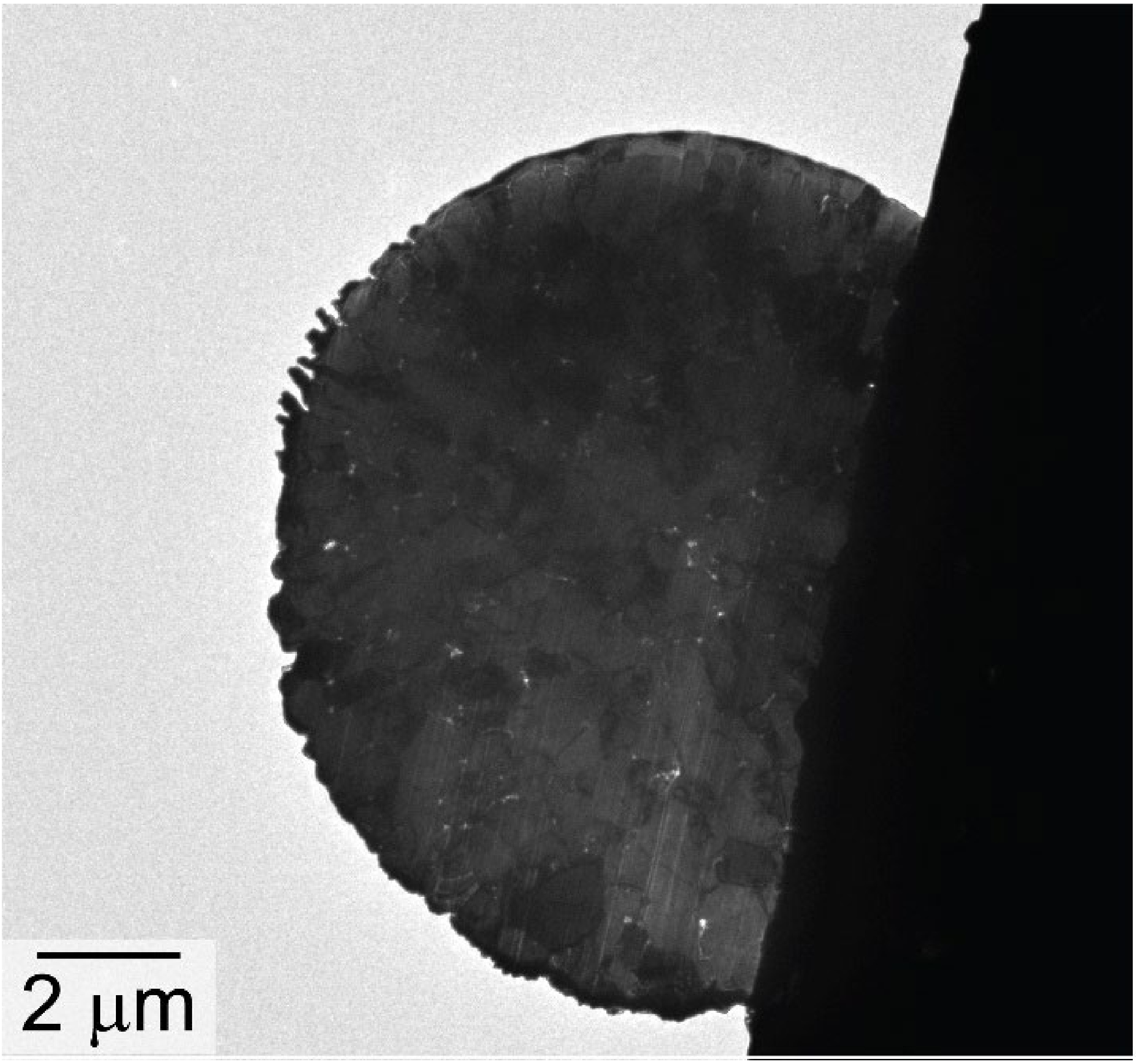

Figure S7. A high-magnification TEM image of a cross-section of a single NCA particle, supported on a finger of a half-moon TEM grid. This cross-section was prepared by FIB milling directly upon the TEM grid after assembly of the particle at an air-liquid interface and transfer to the grid by a custom workflow.

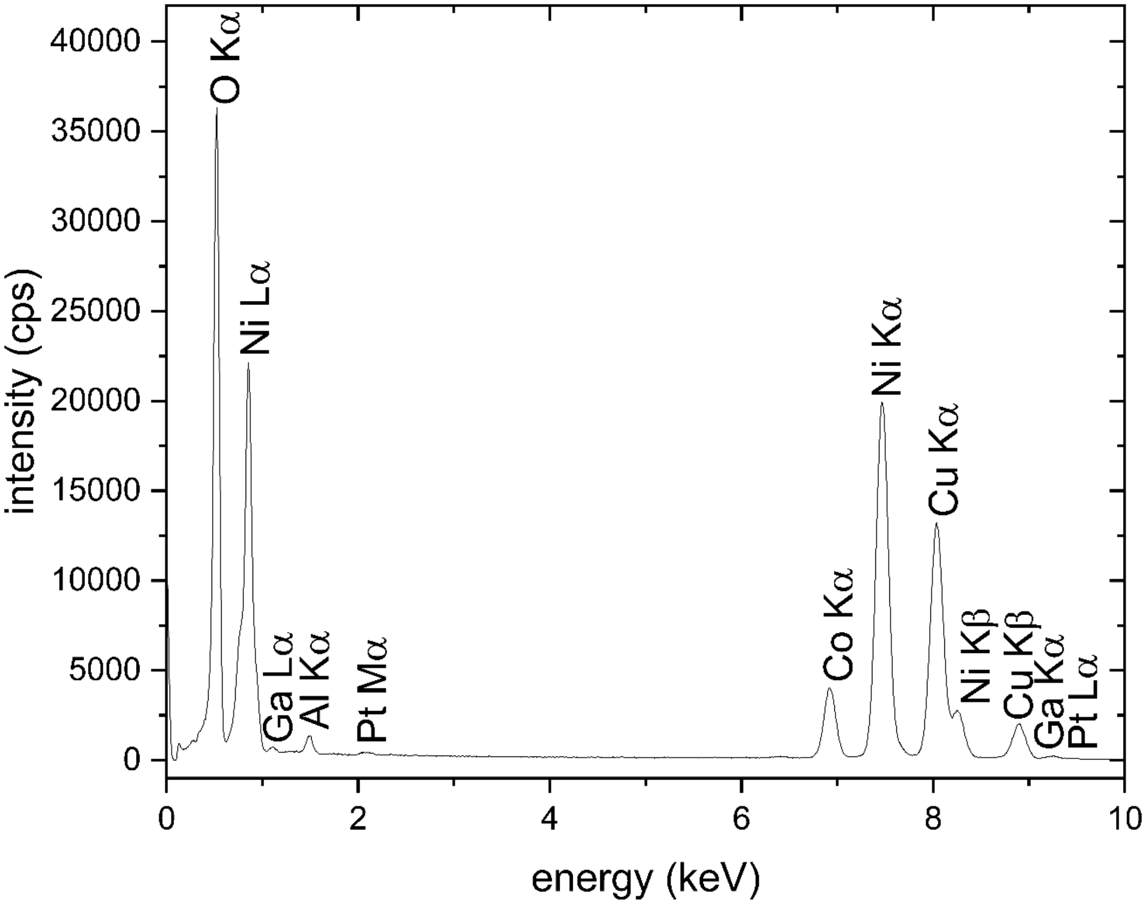

Figure S8. A representative energy dispersive X-ray spectroscopy (EDS) spectrum of the thin section of an NCA particle prepared by FIB techniques as depicted in Figures 1 and 3 of the main text.

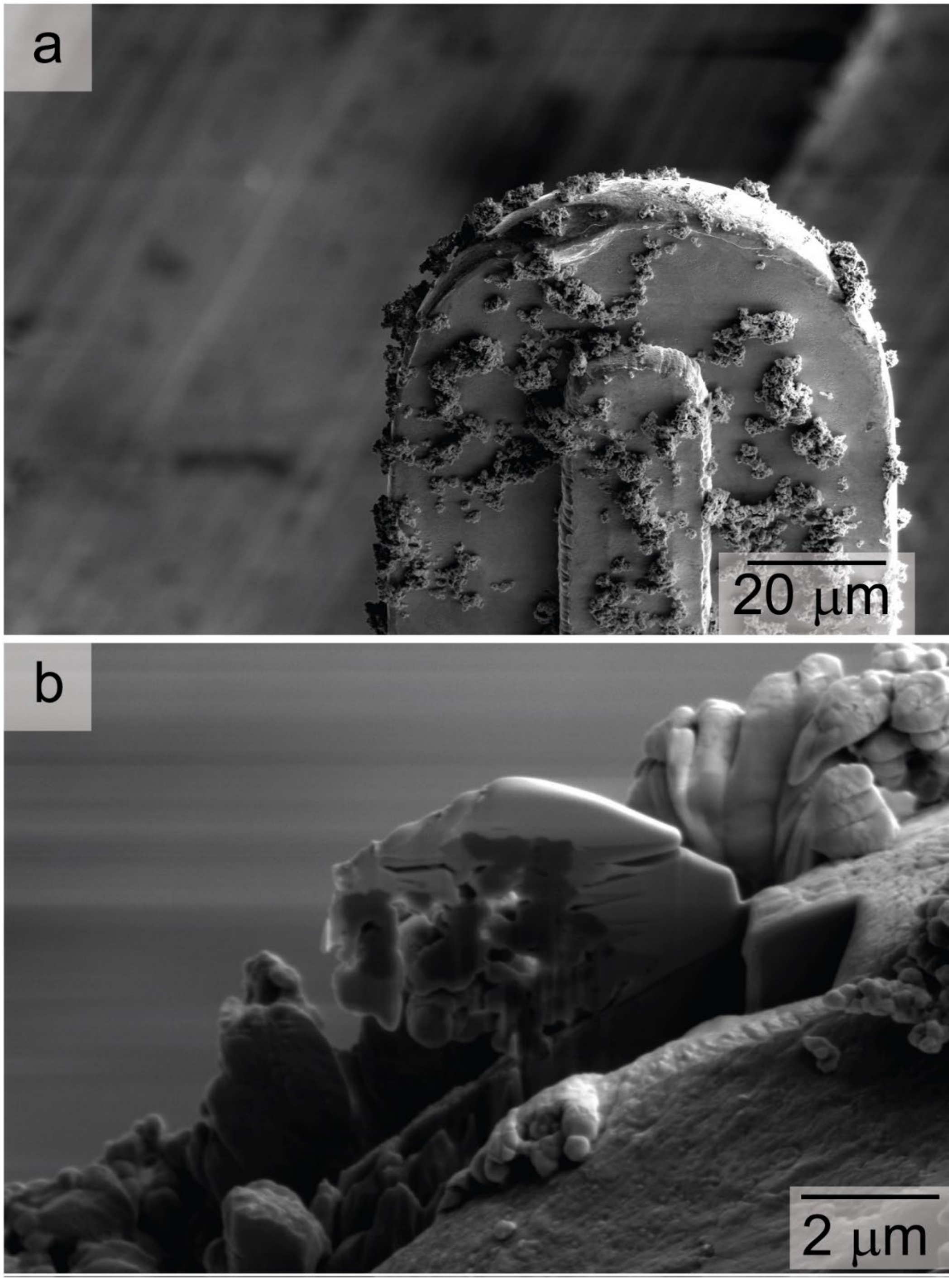

Figure S9. Representative SEM images obtained at a tilt angle of 52° for lithium manganese nickel oxide (LMNO) particles supported on a half-moon TEM grid during the preparation of a thin cross-section. These images depict the (a) pristine particles covering the fingers of the half-moon grid, and (b) the particle after Pt protection and FIB milling.

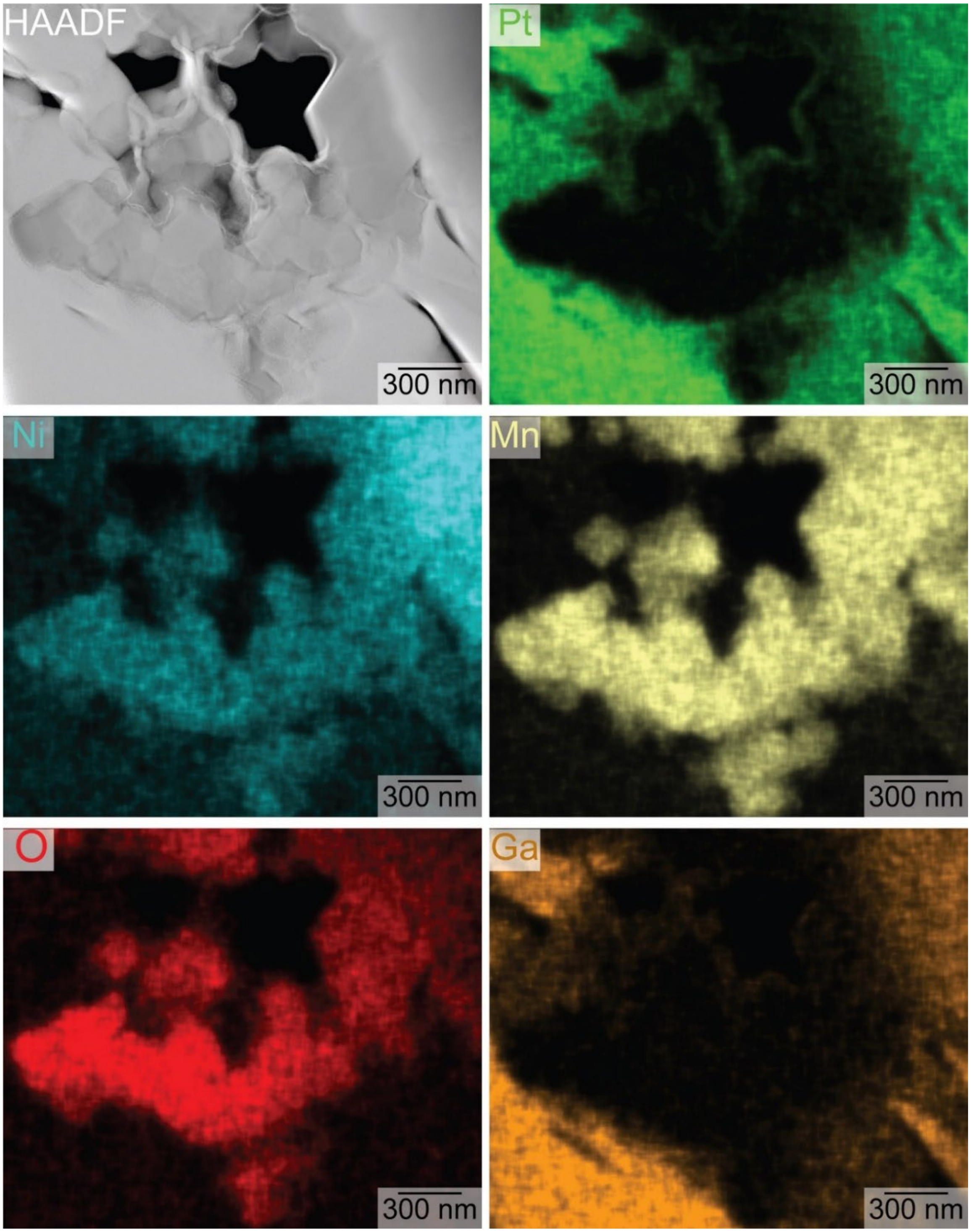

Figure S10. A TEM image obtained by high-angle annular dark-field (HAADF) imaging and EDS maps of the elements (Pt, Ni, Mn, O and Ga) within LMNO particles as seen in this cross-section prepared by FIB methods while the particles were supported on a half-moon TEM grid. The Pt was deposited to protect the sample from the destructive Ga^+ ion beam.

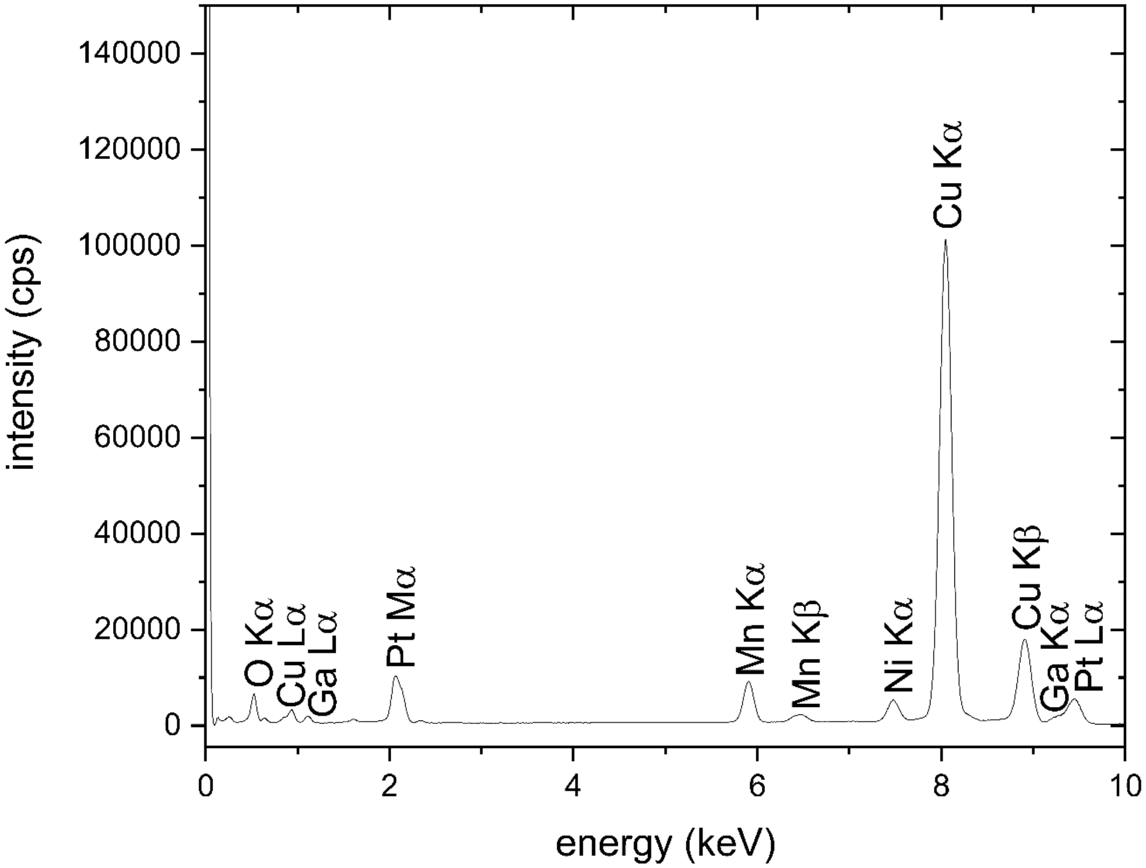

Figure S11. A representative EDS spectrum of the LMNO particles shown in Figure S10. The contribution of copper was from the TEM half-moon grid. The Pt was a protective layer on the LMNO particles, and the Ga resulted from the FIB-based preparation of the cross-section.

Table S1. Peak area ratios as measured by X-ray diffraction (XRD) analysis of NCA materials in their pristine form and after immersion in the respective solvents indicated in the headers.†

ratio of XRD peaks for NCA	pristine NCA	NCA in BuOH	NCA in BuOH/IPA	NCA in BuOH/IPA/H_2O
(003)/(104)	0.903	0.934	0.759	0.852
(101)/(104)	0.568	0.584	0.550	0.540
(006)/(104)	0.110	0.140	0.121	0.120
(102)/(104)	0.207	0.220	0.209	0.205
(105)/(104)	0.194	0.210	0.209	0.198
(107)/(104)	0.194	0.210	0.204	0.198

† BuOH: 1-butanol; IPA: isopropyl alcohol; H_2O: water.
